# Adaptive Decision-Making Policies for Stochastic Electric Vehicle Routing with Time Windows Using Genetic Programming

**DOI:** 10.3390/s26206457

**Published:** 2026-10-12

**Authors:** Alan Đurđević, Nikolina Frid, Marko Đurasević

**Affiliations:** Faculty of Electrical Engineering and Computing, University of Zagreb, 10000 Zagreb, Croatia; alan.durdevic@fer.unizg.hr (A.Đ.); marko.durasevic@fer.unizg.hr (M.Đ.)

**Keywords:** electric vehicle routing problem, sensor-enabled fleet management, stochastic vehicle routing, online routing policies, decision planning, genetic programming, hyper-heuristics

## Abstract

Intelligent transportation systems increasingly rely on electric vehicle fleets operating under uncertain conditions, where routing decisions must adapt to changes in customer demand, service duration, and travel time while satisfying battery, charging, capacity, and time-window constraints. This work extends a genetic programming (GP)-based route generation scheme (RGS) to the stochastic electric vehicle routing problem with time windows (EVRPTW) and positions it as a downstream decision layer for sensor-enabled fleet management: progressively revealed operational conditions update the route state and are translated into online routing actions without repeated optimization. The experiments represent this information flow through controlled stochastic perturbations, enabling reproducible evaluation independently of a particular sensing configuration. The GP representation is extended with stochastic and global-state descriptors, and two capacity-aware RGS variants are introduced. Five RGS variants are evaluated across multiple stochastic scenarios for fleet size, energy consumption, and total tardiness. Limited parallelism performs best for fleet-size minimization, sequential construction for energy minimization, and parallel construction for tardiness minimization. Across the evaluated uncertainty scenarios, the identity of the best-performing RGS family for each objective remained largely unchanged, although absolute performance and dispersion varied non-monotonically. Structural analysis further showed that descriptors related to demand, energy, timing, and fleet state recurred more consistently in the analyzed policies than explicit variability descriptors. With an average online route-construction time of approximately 10 ms per 100-customer instance, the framework provides a computationally lightweight mechanism for converting sensor- and system-derived state updates into adaptive electric-fleet decisions under uncertainty.

## 1. Introduction

Efficient fleet management is a fundamental component of modern transportation and logistics systems. Intelligent transportation systems increasingly aim to improve the efficiency, sustainability, and coordination of transport operations by enabling smarter management of transportation networks and more effective fleet operation [1]. In freight distribution, last-mile delivery, and commercial fleet logistics, operators must continuously assign customer requests to available vehicles and determine efficient service sequences and routes while satisfying operational constraints [2].

These routing and scheduling tasks are commonly formalized as vehicle routing problems (VRPs), which provide a central optimization framework for assigning customers to vehicles and constructing feasible routes under operational constraints [3]. The practical relevance of VRPs arises from a wide range of applications in transportation, logistics, and production systems [4]. Over time, numerous variants have been developed to better reflect real-world requirements, including the vehicle routing problem with time windows (VRPTW), pickup-and-delivery settings, and stochastic or dynamic formulations [5,6,7].

In the context of sustainable transportation, electric vehicle routing problems (EVRPs) have received increasing attention [8,9,10]. In these problems, routing decisions must consider not only customer service requirements, but also battery capacity, charging time, and charging infrastructure constraints. In particular, logistics systems using electric vehicles must explicitly address limited driving ranges and the need for recharging along routes, which is especially critical in mid- and long-haul operations [11].

An important development in EVRP research is the electric vehicle routing problem with time windows (EVRPTW) introduced in [12], which extends the classical Solomon benchmark framework [13] and has become the standard reference model. Early and foundational EVRPTW approaches predominantly considered deterministic settings, where customer demand, travel times, and service durations were assumed to be known in advance [14,15,16]. However, practical transportation systems operate under inherent uncertainty, where customer requests, travel times, energy consumption, and service durations may vary throughout route execution, significantly affecting operational decisions. Such sources of uncertainty have long been recognized in the stochastic VRP literature [17,18], and their incorporation into EV routing remains an active area of research [19].

Existing research has addressed this uncertainty through optimization-based techniques such as stochastic programming and simulation-based optimization [20,21]. These methods can effectively represent stochastic behavior, but they often require repeated optimization or extensive scenario evaluation. Their application may therefore become challenging when operational conditions change during route execution and routing decisions must be generated rapidly.

At the same time, modern fleet-management systems increasingly obtain operational-state information from heterogeneous sensing and communication sources. GPS-enabled probe vehicles and connected or automated vehicles provide position, speed, and surrounding-traffic observations that support real-time traffic monitoring and traffic-state estimation [22,23,24], while battery-management systems use onboard measurements to estimate battery state and support energy-aware operation [25]. Recent work has also combined probe-vehicle trajectories with stationary-sensor data to estimate turning movements, queue lengths, and traffic density in real time [26]. Together with load and service-status information from fleet-information systems, these data provide progressively updated information about the operational state. Their availability does not eliminate uncertainty, but allows changing conditions to be observed during route execution and incorporated into subsequent decisions. This creates a need for routing methods capable of converting such information into adaptive decisions with sufficiently low latency.

Accordingly, routing is increasingly treated not only as a static planning task but also as an online decision-making process based on adaptive policies. This has motivated an alternative line of research concerned with learning routing policies that can be executed directly as operational conditions change. Representative examples include reinforcement learning [27,28] and, more recently, GP-based hyper-heuristic approaches for the EVRPTW [29]. The latter evolve routing policies through a route generation scheme (RGS) rather than relying on iterative solution improvement, enabling fast and adaptive route construction. However, existing GP-based approaches have been developed under deterministic assumptions, leaving a gap between stochastic EV routing and GP-based policy generation.

This paper addresses this gap by extending the GP-based RGS framework to stochastic EVRPTW while retaining the standard Schneider-based benchmark structure. Simultaneous uncertainty in customer demand, travel time, and service duration is modeled through controlled stochastic realizations that provide a technology-agnostic representation of the operational updates that would be progressively supplied by sensing and fleet-information systems. This abstraction enables systematic and reproducible evaluation of the sensing-to-decision process across different uncertainty sources and levels without tying the routing policy to a particular data-acquisition configuration. The framework further introduces stochastic extensions of the route generation scheme and expands the GP representation with descriptors supporting adaptive routing decisions under uncertainty. The objective of the study is therefore to determine whether lightweight GP-evolved constructive policies can use progressively revealed operational information to support fast, objective-specific routing decisions under simultaneous demand, service-time, and travel-time uncertainty.

The main contributions of this work are as follows:

- A technology-agnostic sensing-to-decision formulation of stochastic EVRPTW is developed through a controlled stochastic adaptation of the Schneider benchmark. Simultaneous and progressively revealed variability in demand, service time, and travel time provides a reproducible representation of operational updates without dependence on a particular sensing configuration.
- The GP–RGS framework is extended at both the route-construction and policy-representation levels. The proposed *Semi-parallel-B* and *Parallel-B* variants introduce best-of-*K*, capacity-aware vehicle selection, while the priority-function representation incorporates stochastic, global-state, and objective-specific descriptors.
- A systematic evaluation across multiple uncertainty scenarios and optimization objectives combines performance comparison, statistical testing, greedy reference heuristics, and structural and semantic analysis of the evolved policies. This analysis characterizes objective-dependent differences among the RGS variants and identifies recurrent decision structures, including usage patterns of operational-state and explicit variability descriptors.

The remainder of this paper is organized as follows. Section 2 reviews related work on electric vehicle routing and GP-based hyper-heuristics. Section 3 presents the proposed methodology, including the stochastic problem setting, the routing policy framework, and the GP-based approach for evolving priority functions. Section 4 describes the experimental setup and reports the computational results across different objectives and stochastic scenarios. Section 5 discusses the observed behavior of the routing variants and their interaction with uncertainty. Finally, Section 6 concludes the paper and outlines directions for future research.

## 2. Related Work

Most existing EVRPTW research assumes deterministic problem parameters and relies on metaheuristic optimization methods, particularly adaptive large neighborhood search (ALNS) [14,30,31], iterative local search (ILS) [15], and variable neighborhood search (VNS) [16]. In addition, exact and hybrid methods have also been proposed, including column generation and branch-and-price-based approaches [32,33,34,35,36,37].

In contrast to deterministic formulations, stochastic vehicle routing problems explicitly model uncertainty in key parameters. Early work [38] established fundamental properties of routing with stochastic demands, including the distinction between full and split delivery and different modes of information revelation. A comprehensive overview of stochastic routing variants, including stochastic customers, stochastic demands, and stochastic travel times, is provided in [17]. Similarly, stochastic VRP is classified into three main classes: stochastic customers, stochastic demands, and stochastic times [18]. The authors also discuss solution approaches such as chance-constrained programming and stochastic programming with recourse. These frameworks typically follow a two-stage structure, where an initial solution is constructed before uncertainty is realized, followed by corrective actions once stochastic information becomes available.

Several works have addressed specific stochastic VRP variants. In [39] routing problems with stochastic travel and service times were studied, while in [40] the authors investigated stochastic travel times using branch-and-cut approaches combined with sampling. For stochastic demands, exact and decomposition-based methods were proposed in [41,42], and reinforcement learning approaches were explored in [43].

Compared with the classical VRP, uncertainty-aware extensions of EVRP and EVRPTW remain relatively limited and generally focus on specific sources of uncertainty. In [21], the authors studied EVRP with stochastic demands and dynamic remedial measures using a mixed-integer programming framework, while [20] introduced stochastic waiting times at charging stations. In [44], uncertain travel time is represented through mean–standard-deviation terms, and an EVRPTW with partial recharging and capacitated charging stations is solved using a Lagrangian-relaxation and ADMM-based decomposition framework. Demand uncertainty and environmental effects such as ambient temperature were incorporated into a stochastic electric vehicle location-routing problem in [19]. Energy-consumption uncertainty has also been addressed through robust optimization in [45] and through two-stage stochastic programming with recourse policies in [46]. Taken together, these studies have advanced uncertainty-aware electric vehicle routing, but simultaneous uncertainty in customer demand, travel time, and service time remains comparatively underexplored.

A complementary research direction focuses on learning routing policies that can be executed directly during route construction. Representative recent learning-based approaches include safe reinforcement learning for dynamic stochastic EV routing [27], Q-learning for capacitated electric vehicle routing [47], and deep reinforcement learning combined with graph convolutional networks for electric vehicle routing with stochastic customer requests [48]. Attention- and transformer-based approaches have also recently been proposed for EVRPTW [49,50]. Hyper-heuristics offer another policy-learning paradigm by generating or learning reusable decision strategies that can be directly applied during solution construction, rather than repeatedly solving optimization models for individual problem instances [51,52]. In particular, constructive hyper-heuristics incrementally build solutions through decision policies, making them well suited for dynamic routing environments requiring rapid online decisions. However, designing effective constructive policies remains non-trivial, and consequently such approaches have received considerably less attention than improvement-based metaheuristics [53].

Genetic programming (GP) [54] has been increasingly used for the automatic design of heuristics, particularly in scheduling [55,56] and graph-based optimization problems [57,58]. Its application to vehicle routing, however, remains relatively limited. Jacobsen et al. [59] first applied GP to evolve heuristics for a dynamic VRP with online customer arrivals, demonstrating clear improvements over manually designed heuristics. Similar findings were reported in [60] for varying levels of dynamic customer arrivals. GP has also been applied to the VRP with zone-based pricing, where evolved heuristics outperformed a genetic algorithm and achieved performance competitive with branch-and-price optimization [58].

One of the first GP-based hyper-heuristic frameworks for the EVRPTW was proposed in [29], where GP was combined with a route generation scheme (RGS) to evolve routing policies instead of directly optimizing complete solutions. The framework was subsequently enhanced through surrogate-assisted learning to improve GP efficiency and convergence [61]. These studies establish GP as a viable mechanism for routing-policy generation but address deterministic EVRPTW formulations only.

## 3. Materials and Methods

### 3.1. Electric Vehicle Routing Problem with Time Windows

EVRPTW extends the classical VRP by considering electric vehicles and time-constrained service at customers. The formulation introduced by [12] has become the standard reference model, and most existing EVRPTW studies are based on this framework. Each customer is associated with a predefined service time window, while routing decisions are constrained by limited battery capacity, energy consumption, and recharging requirements. In contrast to conventional vehicles, battery recharging is significantly slower than refueling, which introduces additional temporal constraints and complicates route planning. As a result, routing decisions involve not only customer assignment and sequencing, but also battery feasibility, charging scheduling, and time-window considerations.

The problem is defined on a fully connected symmetric graph G=(N,L), where nodes represent the depot, customers, and charging stations. A single depot is assumed, serving as both the start and end location for all routes. Each link (i,j)∈L is associated with a distance $dist_{ij}=dist_{ji}$, which determines the corresponding travel time, while energy consumption is assumed proportional to traveled distance. To formally define the EVRPTW setting considered in this work, the notation listed in Table 1 is used.

Each customer must be serviced exactly once by a single vehicle, prohibiting split deliveries. Service at customer $i\in N_{c}$ requires service duration $s_{i}$ and is associated with the time window $\left[a_{i},b_{i}\right]$. Vehicles arriving before $a_{i}$ wait until service can begin, whereas service completed after $b_{i}$ contributes to tardiness.

A homogeneous fleet of vehicles is available at the depot. Each vehicle has cargo capacity *C*, battery capacity *Q*, and travels at nominal speed *v*. Vehicles depart from the depot fully loaded and fully charged. Cargo capacity decreases as customers are served and cannot be replenished during route execution, while battery consumption is assumed proportional to traveled distance.

Charging stations allow vehicles to recharge their batteries during route execution. A full recharging strategy is assumed, meaning that each charging visit restores the battery to full capacity. Charging time is modeled linearly using charging rate *g*, such that recharging duration is proportional to the replenished energy amount. Charging stations are assumed to have unlimited capacity, allowing simultaneous charging of multiple vehicles.

Three optimization objectives are considered independently: minimization of fleet size, total energy consumption, and total tardiness, subject to customer-service, cargo-capacity, battery, charging, and time-window constraints.

The total tardiness objective is defined as

(1) $$T_{tot}=\sum_{i\in N_{c}}T_{i}.$$

Total energy consumption is computed as

(2) $$E_{tot}=\sum_{\left(i,j\right)\in L_{used}}r\cdot dist_{ij},$$

where $L_{used}$ denotes the set of traversed route links and *r* is the energy-consumption rate.

The fleet-size objective is defined as

(3) $$V_{tot}=\left|V_{used}\right|,$$

where $V_{used}$ denotes the set of vehicles used to service all customers.

### 3.2. Stochastic Extension and Information Revelation

In this work, a stochastic extension of the EVRPTW with *soft* time windows is considered. Uncertainty is introduced through multiplicative random factors applied independently to customer demand, service time, and vehicle speed, denoted by $\lambda^{\left(d\right)}$, $\lambda^{\left(s\right)}$, and $\lambda^{\left(v\right)}$, respectively. These sources of uncertainty follow established stochastic VRP formulations [17,18,39], where variability in demand, service time, and travel time forms the standard modeling basis. Travel-time uncertainty is implemented indirectly via stochastic vehicle speed, preserving the network structure while inducing variability in traversal times.

The variability of each stochastic factor is controlled through a normalized variability parameter denoted by CV, while preserving the deterministic parameter as the expected value of the corresponding stochastic variable. Consequently, the deterministic instance represents the mean-case scenario, whereas increasing CV introduces progressively stronger stochastic perturbations around the nominal values. In this study, values between CV=0.1 and CV=0.3 are used to represent moderate to pronounced uncertainty levels.

Two distributions are considered in order to model qualitatively different uncertainty regimes. The first is a bounded symmetric uncertainty model implemented using a uniform distribution,

(4) λ∼U[1−CV,1+CV],

which represents controlled fluctuations around the nominal value. The second is a positively skewed uncertainty model implemented using a lognormal distribution,

(5) $$\sigma =\sqrt{\ln(1+CV^{2})},\;\mu =-\frac{\sigma^{2}}{2},\;\lambda ∼\mathrm{Lognormal}\left(\mu ,\sigma^{2}\right),$$

allowing strictly positive and asymmetric deviations with occasional larger realizations, better reflecting rare but substantial operational disruptions. Such parametric uncertainty models are commonly used in stochastic VRP and EVRP studies to provide controlled and reproducible uncertainty conditions [17,18,21].

The stochastic parameters are defined as

(6) $${\tilde{d}}_{i}=d_{i}\lambda^{\left(d\right)},\;{\tilde{s}}_{i}=s_{i}\lambda^{\left(s\right)},\;\tilde{v}=v\lambda^{\left(v\right)},$$

where $\lambda^{\left(d\right)}$, $\lambda^{\left(s\right)}$, and $\lambda^{\left(v\right)}$ are mutually independent random variables representing demand, service-time, and speed variability, respectively. Although these uncertainty sources may be correlated in practice, correlated stochastic processes are not considered in this work. The independence assumption enables the individual and combined effects of the principal uncertainty sources to be isolated within a controlled and computationally tractable experimental setting.

Vehicle travel times are computed using the realized speed as

(7) $${\tilde{t}}_{ij}=\frac{dist_{ij}}{\tilde{v}}=\frac{dist_{ij}}{v\lambda^{\left(v\right)}}.$$

Under the distance-proportional energy model in Equation (2), a change in realized speed alters the traversal time but not the energy required to traverse a fixed link. Thus, stochastic speed represents travel-time uncertainty and does not introduce a separate speed-dependent energy component.

A new realization of $\lambda^{\left(v\right)}$ is independently sampled for each vehicle movement, inducing stochastic travel times throughout route execution. In contrast, the realized customer demand ${\tilde{d}}_{i}$ and service time ${\tilde{s}}_{i}$ become known only upon arrival at customer *i*, reflecting the standard assumption of online information revelation in stochastic VRP models [41,42,43].

Beyond introducing variability, this formulation defines how operational information becomes available during route execution. Before a vehicle movement or customer visit, the corresponding stochastic quantities remain unresolved and are represented by their probability distributions. Once the event occurs, the realized values become available and update the vehicle and route state used in subsequent decisions. Controlled stochastic realizations therefore provide a technology-agnostic representation of both changing operational conditions and the progressive arrival of new information.

In an operational fleet-management system, analogous state updates could be obtained from onboard sensing, traffic-monitoring, and fleet-information systems, while the corresponding probability models could be calibrated from historical or streaming operational data. By abstracting from a particular data-acquisition configuration, the stochastic formulation enables the sensing-to-decision process to be evaluated systematically and reproducibly across different uncertainty sources and levels.

### 3.3. Routing Policy

Because information becomes progressively available in the stochastic setting defined above, routes constructed entirely from nominal parameters may become suboptimal or infeasible as execution proceeds. An adaptive routing policy can instead respond to realized conditions by using the updated vehicle and route state when making subsequent decisions. The proposed framework therefore provides the adaptive decision component of the sensing-to-decision process: routing decisions are generated sequentially from the system state and stochastic information available at each decision point, rather than by repeatedly solving a complete routing optimization problem after every update.

The framework builds upon the GP-based routing-policy approach introduced for the deterministic EVRPTW in [29], extending it with stochastic route-generation mechanisms and additional descriptors to support adaptive decision-making under uncertainty. The resulting routing policy consists of two components:

1. **Route Generation Scheme (RGS)**, which defines how routes are incrementally constructed;
2. **Priority Function (PF)**, which determines which customer should be selected next at each decision point.

**Figure 1:**
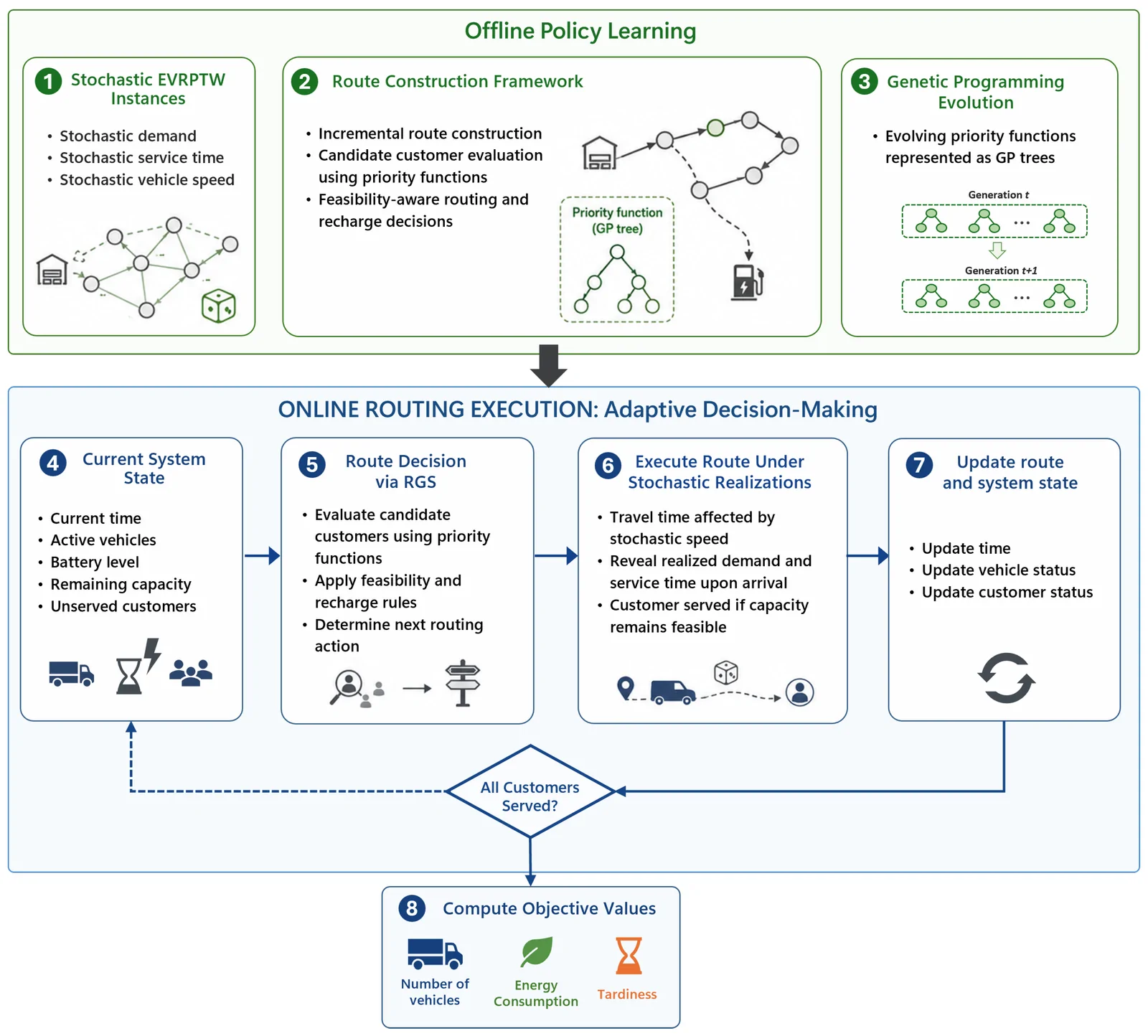
Overview of the proposed GP-based routing framework for stochastic EV routing. Offline training evolves priority functions represented as GP trees, while online execution adaptively constructs routes as stochastic information is revealed and the system state is updated. Solid arrows indicate the main processing flow, while the dashed feedback arrow indicates repetition of the online decision cycle until all customers are served.

Figure 1 provides an overview of the proposed framework, distinguishing the offline evolution of priority functions from the online state-update and adaptive route-construction process. The route-construction process defined by the RGS is detailed in the following section, while the representation and learning of the priority function are described in Section 3.5.  

### 3.4. Route Generation Scheme

RGS defines the mechanism by which vehicle routes are constructed. In this study, five variants are considered: the Serial, Semi-parallel, and Parallel schemes adopted from [29], along with two extensions proposed in this work, namely Semi-parallel-B and Parallel-B, where the suffix B denotes the best-of-K vehicle-selection rule introduced here.

#### 3.4.1. Common Feasibility and Operational Rules

Energy feasibility is verified before each movement decision. A vehicle may proceed to a destination only if it has sufficient energy to (i) reach the destination and (ii) subsequently reach the nearest charging station, preventing infeasible states without access to recharging. If this condition is not satisfied, the vehicle first travels to a feasible charging station, recharges, and then continues its route. Charging stations are selected by minimizing the total energy required to travel from the current location to the station and from the station to the intended destination.

Under stochastic demand, the realized demand of a selected customer may exceed the vehicle’s remaining capacity. In such cases, the service attempt is aborted, the vehicle returns to the depot (via a charging station if necessary), and the route is finalized. The customer remains unserved and is reconsidered later. This mechanism ensures feasibility under stochastic realizations and is applied implicitly in all RGS variants, although omitted from Algorithm 1 for clarity.
**Algorithm 1** Unified RGS framework**Require:**
variant ∈ {Serial, Semi-parallel, Parallel, Semi-parallel-B, Parallel-B}
  1:$\mathrm{UC}\leftarrow N_{c}$                     ▹ set of unserved customers  2:Routes←∅  3:V← InitializeActiveVehicles (*variant*, *LB*)  4:vehicle←null  5:**while** UC≠∅ **do**  6:     **if** vehicle=null **then**  7:           vehicle← SelectOrActivateVehicle (*variant*, *V*)  8:     **end if**  9:     customer← select a customer from UC using the PF10:     **if** vehicle has sufficient remaining capacity for nominal demand of customer **then**11:           destination←customer12:     **else**13:           destination←014:     **end if**15:     **if** vehicle does not have sufficient battery charge to reach destination and then the nearest charging station in $N_{s}$ **then**16:           chargingStation← select a charging station from $N_{s}$17:           move vehicle to chargingStation18:           recharge vehicle19:     **end if**20:     move vehicle to destination21:     **if** destination∈UC **then**22:           serve customer23:           UC←UC∖{customer}24:     **else**25:           add the completed route of vehicle to Routes26:           V← UpdateActiveVehicles (*variant*, *V*, *vehicle*)27:           vehicle←null28:     **end if**29:**end while**30:**return** Routes


Time windows are modeled as soft because stochastic service times and travel speeds can make hard-window routes infeasible and require repair mechanisms outside the scope of this study. Late service is therefore allowed and penalized through the tardiness objective, consistent with practical settings in which limited delays are tolerated.

In the Semi-parallel and Parallel variants, LB denotes a capacity-based lower bound on the required number of vehicles, computed as

(8) $$LB=\left\lceil \frac{\sum_{i\in N_{c}}d_{i}}{C}\right\rceil ,$$

where $N_{c}$ is the set of customer nodes. This bound represents the minimum number of vehicles required to satisfy total nominal customer demand under cargo-capacity constraints. Because the stochastic demand multipliers are centered at one, the nominal aggregate demand is equal to its expected value. The resulting LB therefore represents a non-anticipative mean-demand capacity estimate based on the information available before demands are realized. Importantly, LB determines only the number of initially active vehicles and does not constrain the final fleet size; additional vehicles are activated during route construction according to the rules of each RGS variant. Depending on a particular realization, the eventual capacity requirement may be either above or below the initial estimate.

#### 3.4.2. Baseline RGS Variants

The Serial, Semi-parallel, and Parallel RGS variants adopted from [29] differ in how vehicles are activated and extended during route construction. In the Serial scheme, routes are constructed sequentially by activating one vehicle at a time. In contrast, the Semi-parallel and Parallel schemes extend multiple routes concurrently, initially activating LB vehicles. The Semi-parallel variant selects the earliest available vehicle for extension and may switch to sequential construction if all active vehicles complete their routes. The Parallel variant maintains continuous parallelism by immediately activating a new vehicle whenever one completes its route. All variants follow the same route construction logic: routes are incrementally extended by selecting an unserved customer, verifying capacity and energy feasibility, and either continuing the route or returning the vehicle to the depot. The differences between variants lie solely in vehicle initialization and selection policies.

#### 3.4.3. Extended RGS with Candidate Set Selection

The proposed Semi-parallel-B and Parallel-B variants extend their respective base schemes through best-of-K vehicle selection. Instead of selecting the earliest available vehicle directly, the *K* earliest available vehicles are considered, and the vehicle with the largest remaining capacity is chosen. In this study, *K* is set to 3, providing a small candidate set that limits premature capacity saturation while keeping the selection process local and computationally efficient.

#### 3.4.4. Unified RGS Framework

All variants described above can be expressed within a single unified route construction framework. The variants differ only in vehicle initialization, selection, and update rules, while the underlying route construction logic remains identical. This unified procedure is presented in Algorithm 1, with variant-specific rules summarized in Table 2.

### 3.5. Genetic Programming for Evolving Priority Functions

While the RGS defines how routes are constructed, the quality of the routing policy depends on the rule used to select the next customer at each decision point. In this work, this rule is represented by a priority function (PF), which assigns a numerical score to each unserved customer, and the customer with the highest score is selected.

The PF is automatically evolved using Genetic Programming (GP), which constructs decision rules by combining problem-specific features through mathematical operators [54,62]. Each GP individual represents a candidate PF encoded as an expression tree, where terminal nodes correspond to features of the current system state and internal nodes represent mathematical operators.

During route construction, the expression tree is evaluated for each candidate customer. Most terminals are computed from the current system state or nominal parameter values and therefore remain unchanged across repeated evaluations. The exceptions are ${\mathrm{BestOtherETA}}_{i}$ and SlackSelf(i), which depend on stochastic estimates and are computed using independently sampled realizations. Each stochastic evaluation can consequently produce a different customer ranking and a different highest-priority customer. The priority function is evaluated five times at each decision point, and the customer selected most frequently across the five evaluations is chosen. If multiple customers receive the same highest number of votes, the customer with the lowest identifier is selected.

At a fixed decision state, let $p_{i}$ denote the probability that customer *i* receives the highest priority under an independently sampled realization. Assuming a unique modal customer, let $i^{*}=\arg\max_{i}p_{i}$. For $n_{\mathrm{vote}}$ independent evaluations, let $\Delta_{j}=p_{i^{*}}-p_{j}>0$ denote the probability gap between the modal customer and a competing customer *j*. Applying Hoeffding’s inequality and a union bound gives

(9) $$\mathrm{Pr}\;\left(\hat{i}\neq i^{*}\right)\leq \sum_{j\neq i^{*}}\exp\;\left(-\frac{n_{\mathrm{vote}}\Delta_{j}^{2}}{2}\right),$$

where $\hat{i}$ is the empirical plurality choice obtained using the deterministic tie-breaking rule described above. Thus, increasing $n_{\mathrm{vote}}$ reduces the probability that the empirical plurality choice differs from the modal decision, while the stochastic priority-function evaluation cost grows linearly with $n_{\mathrm{vote}}$. The practical benefit depends on the state-specific probability gaps: few evaluations may be sufficient when one customer is clearly preferred, whereas considerably more evaluations may be required when several customers have similar selection probabilities.

In this study, $n_{\mathrm{vote}}=5$ was fixed a priori as a small fixed stochastic evaluation budget, consistent with the use of small fixed sampling batches in related stochastic GP hyper-heuristics [57,63,64], although those studies apply sampling at the fitness-evaluation rather than decision level. The value was applied uniformly across all RGS variants, objectives, training runs, and test conditions and is not claimed to be theoretically optimal. Relative to the selected value, $n_{\mathrm{vote}}=3$ would reduce the stochastic priority-function evaluation cost by 40% but would be expected to produce less stable empirical plurality decisions. Conversely, $n_{\mathrm{vote}}=10$ would double this component of the execution cost while generally reducing sampling-induced decision variability. The magnitude of the corresponding performance change cannot be determined from Equation (9), because the probability gaps vary across decision states and route trajectories.

Because $n_{\mathrm{vote}}$ is used during GP fitness evaluation, it forms part of the policy-development configuration: changing it can alter customer selections, complete route trajectories, individual fitness values, and evolutionary selection. A controlled sensitivity analysis would therefore require independent policy evolution under each considered voting budget. Applying an alternative value only after evolution would instead assess the deployment robustness of policies under a changed voting budget, rather than provide a controlled sensitivity analysis of the policy-development configuration. Consequently, the comparative conclusions reported here are conditional on the common fixed budget $n_{\mathrm{vote}}=5$.

The GP procedure follows a standard generational evolutionary scheme, where a population of candidate PFs is iteratively improved through selection, crossover, and mutation. The initial population consists of 200 expression trees generated using the full method [54], with a depth of five. During evolution, the tree depth is limited to $2^{8}-1$, and individuals exceeding this limit are considered invalid and removed from the population. The evolutionary process runs for 1000 generations. The population size and number of generations were retained from the deterministic GP–RGS framework [29] to keep the experimental setup comparable, while the remaining configuration follows standard GP practice [54]. The general workflow is shown in Algorithm 2, and the configuration parameters are summarized in Table 3. The implementation is based on the *Jenetics* (https://jenetics.io/) evolutionary computation library (version 8.3.0).
**Algorithm 2** Descriptive evolutionary workflow in Jenetics  1:Generate the initial population $P_{0}$  2:Evaluate the fitness of all individuals in $P_{0}$  3:**while** the termination criterion is not satisfied **do**  4:      Increment the generation counter: g←g+1  5:      Select the survivor population $S_{g}$ from the previous population $P_{g-1}$  6:      Select the offspring population $O_{g}$ from the previous population $P_{g-1}$  7:      Apply genetic alterations (crossover and mutation) to $O_{g}$  8:      Remove invalid individuals from $S_{g}$ and $O_{g}$  9:      Construct the new population $P_{g}$ by combining the filtered survivors and offspring10:      Evaluate the fitness of all individuals in $P_{g}$11:**end while**

#### 3.5.1. Terminal and Function Nodes

The primitive set builds upon the terminals and functions proposed in [29], which are retained to preserve the original EVRPTW routing policy framework. To address the stochastic setting considered in this work, the terminal set is extended with stochastic descriptors (Var_Dni, Var_Tij, Var_Sni) intended to capture expected variability in customer demand, travel time, and service time, as well as global-state and coordination descriptors (UC, DsumUC, CsumV, $BestOtherETA_{i}$) intended to provide information about the remaining workload, available vehicle resources, and interactions between concurrently active vehicles. In addition, Slack_TW is included to provide information about the remaining time-window slack available for servicing a customer.

For objective-specific optimization, CminV is introduced for the vehicle minimization objective to capture the minimum remaining capacity among active vehicles, while SlackSelf(i) is introduced for tardiness minimization to capture the available temporal slack of the currently considered vehicle for customer *i*, computed as the difference between the customer due time and the estimated vehicle arrival time.

Due to the online nature of the considered setting, future realized demand, service time, and travel time are not available during customer selection. Accordingly, terminals depending on unrevealed stochastic quantities are generally evaluated using nominal parameter values. The exceptions are ${\mathrm{BestOtherETA}}_{i}$ and SlackSelf(i), which are computed using sampled stochastic estimates as described above. Terminals representing already observed vehicle and system states use the currently available realized information.

The complete set of terminal nodes is summarized in Table 4, while their relevance and usage patterns within evolved routing policies are further analyzed in Section 4.7.

The function nodes used in the GP representation are summarized in Table 5. The first row contains binary operators, and the second row contains unary operators. To ensure numerical stability during tree evaluation, the operators *div*, *log*, and *sqr* are implemented as safe functions. Specifically, *div* returns 0 when the denominator is close to zero, while *log* and *sqr* return 0 for non-positive arguments.

#### 3.5.2. Computational Characteristics

The computational cost of applying an evolved GP–RGS policy can be characterized in terms of the route-construction process. Let *n* denote the number of customers, *m* the number of charging stations, *D* the number of route-extension decisions, $u_{d}$ the number of unserved customers at decision *d*, $n_{\mathrm{vote}}$ the decision-level voting count, and |T| the number of nodes in the priority-function tree. At decision *d*, the priority function is evaluated $n_{\mathrm{vote}}$ times for each of the $u_{d}$ unserved customers. Because one tree evaluation requires O(|T|) operations, the total priority-evaluation cost of one route-construction execution is

(10) $$O\left(n_{\mathrm{vote}}\left|T\right|\sum_{d=1}^{D}u_{d}\right).$$

Since $u_{d}\leq n$, this cost is bounded above by $O(n_{\mathrm{vote}}|T|nD)$. If the number of route-extension decisions grows linearly with the number of customers, the priority-evaluation component becomes $O(n_{\mathrm{vote}}|T|n^{2})$. Retaining *D* explicitly in Equation (10) also accounts for additional decisions caused by route completion or unsuccessful service attempts under stochastic demand.

Charging-station selection contributes at most O(mD) when the available stations are inspected at each relevant decision. The best-of-*K* vehicle-selection rule used by the proposed B-variants contributes O(KD). The total online time complexity of applying an evolved policy can therefore be expressed as

(11) $$O\left(n_{\mathrm{vote}}\left|T\right|\sum_{d=1}^{D}u_{d}+mD+KD\right).$$

Table 6 summarizes how the principal problem- and policy-related quantities contribute to online execution cost.

The working state maintained during route construction is linear in the numbers of customers, charging stations, active vehicles, constructed movements, and priority-function nodes. If *v* denotes the number of active vehicles, the auxiliary working memory is O(n+m+v+D+|T|). When a complete distance or travel-time matrix is stored, the instance representation additionally requires $O({\left(n+m\right)}^{2})$ memory.

Offline GP evolution repeatedly invokes the same route-construction procedure. Let *E* denote the total number of candidate priority functions whose fitness is evaluated, $I_{\mathrm{train}}$ the number of training instances, and $n_{\mathrm{fit}}$ the number of stochastic fitness evaluations performed per training instance. The offline training cost scales as

(12) $$O\left(EI_{\mathrm{train}}n_{\mathrm{fit}}C_{\mathrm{route}}\right),$$

where $C_{\mathrm{route}}$ denotes the cost of one route-construction execution, including decision-level stochastic voting. This expression distinguishes the repeated offline cost of policy evolution from the cost of subsequently applying an evolved policy to a new problem instance.

## 4. Results

### 4.1. Experimental Protocol

The experiments were conducted on a subset of Schneider’s benchmark instances [12] for the EVRPTW. In total, 48 instances were used, each consisting of 100 customers, 21 charging stations, and one depot. These instances represent the largest and most demanding problem scale available in the Schneider benchmark. This scale provides a more substantive evaluation of adaptive route construction under simultaneous uncertainty than the smaller benchmark subsets, which contain comparatively few customer-selection decisions. According to the spatial distribution of customers, the instances are categorized into Clustered (C), Random (R), and Random-Clustered (RC) sets.

Because GP directly optimizes the priority functions on the training instances, evaluation on a disjoint test set is required to determine whether the evolved policies generalize beyond the instances encountered during evolution. The dataset was therefore split into mutually disjoint training and test sets. The training set contains 30 instances (10 C, 10 RC, and 10 R), while the test set contains the remaining 18 instances (6 C, 6 RC, and 6 R). The split is stratified by spatial category, ensuring that all three instance types are represented in both sets. The larger training set exposes GP to diverse spatial configurations, while the balanced test set retains six previously unseen instances from each category. All results reported in this section are obtained on the test set.

During training, each instance was evaluated twice per fitness computation, exposing candidate policies to multiple stochastic realizations during evolution. All GP models were evolved under a fixed stochastic setting in which customer demand, service time, and vehicle speed follow a lognormal distribution with variability parameter CV=0.2. Within the sensing-to-decision interpretation, this fixed distribution provides a controlled counterpart of an uncertainty model that could be calibrated from historical operational data, while repeated evaluations expose candidate policies to different sequences of updates consistent with that model. To account for both stochasticity and the randomness of GP initialization, the entire training procedure was repeated ten times, resulting in ten independently evolved PFs for each RGS–objective configuration. These independent runs enable run-to-run variability to be summarized for each configuration. The GP budget, candidate-set size K, and decision-level voting budget were held fixed throughout the experiments; their sensitivity was not evaluated separately.

Each evolved PF was subsequently evaluated across a range of stochastic test scenarios that systematically vary the distribution type and variability level of demand, service time, and vehicle speed. These scenarios test whether policies evolved under the fixed lognormal CV=0.2 training condition transfer to uncertainty patterns not encountered during evolution. Operationally, this represents a potential mismatch between the uncertainty model used during policy development and the conditions subsequently encountered during deployment. A test scenario is denoted as

$$\left\{\mathrm{distribution}\right\}-\left\{{\mathrm{CV}}_{d}\right\},\left\{{\mathrm{CV}}_{s}\right\},\left\{{\mathrm{CV}}_{v}\right\},$$

where distribution specifies the probability distribution (DET for deterministic, LN for lognormal, and U for uniform), and $CV_{d}$, $CV_{s}$, and $CV_{v}$ denote the variability parameters associated with customer demand, service time, and vehicle speed, respectively. The deterministic scenario DET-0,0,0 corresponds to the standard EVRPTW without stochastic perturbations and serves as an internal baseline, enabling comparison with the original deterministic GP-RGS framework [29] under identical evaluation conditions and isolating the effect of incorporating stochastic information into the routing policy. This abbreviated notation is used consistently throughout the result tables. The considered evaluation scenarios are listed in Table 7.

For each test scenario, every PF is evaluated on all 18 test instances (6 C, 6 RC, and 6 R). To account for stochastic variability, each instance is evaluated six times using independently sampled realizations of demand, service time, and travel time. Each realization represents a distinct execution trajectory in which operational information becomes available at the revelation points defined in Section 3.2. This results in 108 evaluations per PF and scenario and captures variation across both the balanced test set and different sequences of progressively revealed operational conditions. These repetitions form part of the experimental test protocol and are not a parameter of the GP algorithm or routing policy. They preserve the 18 structurally distinct test instances while expanding the evaluated set of stochastic execution trajectories and enabling paired comparisons under independently seeded operational realizations. For a given PF and scenario, the objective values are summed over all 108 evaluations, yielding a single score per PF. The reported results summarize the distribution of these scores across the ten independently evolved PFs using the mean, standard deviation, and minimum value, together with the full distributions shown in the violin plots. This evaluation procedure is applied consistently across all optimization objectives.

Because each independently seeded execution samples one realization of customer demand, service time, and travel time, repeated evaluation provides a Monte Carlo estimate of expected policy performance under the specified stochastic scenario. For a fixed policy, test instance *i*, and stochastic scenario, let $Y_{i,r}$ denote the objective value obtained from execution *r*, and let $n_{\mathrm{test}}$ denote the number of executions per instance. Conditional on the fixed set of I=18 benchmark instances, the mean performance estimator is

(13) $$\hat{\mu }=\frac{1}{In_{\mathrm{test}}}\sum_{i=1}^{I}\sum_{r=1}^{n_{\mathrm{test}}}Y_{i,r}.$$

If the executions are independently seeded and $Y_{i,r}$ has variance $\sigma_{i}^{2}$, the stochastic component of the estimator variance is

(14) $$\mathrm{Var}\left(\hat{\mu }|I\right)=\frac{1}{I^{2}n_{\mathrm{test}}}\sum_{i=1}^{I}\sigma_{i}^{2},$$

where I denotes the fixed benchmark set. Consequently, increasing $n_{\mathrm{test}}$ reduces the variance attributable to stochastic realizations proportionally to $1/n_{\mathrm{test}}$ and its standard error proportionally to $1/\sqrt{n_{\mathrm{test}}}$, while increasing the evaluation cost linearly. The PF-level score reported below is the corresponding unnormalized sum. Because *I* and $n_{\mathrm{test}}$ are identical for all compared policies, it differs from $\hat{\mu }$ only by the common factor $In_{\mathrm{test}}$.

The $n_{\mathrm{test}}$ repetitions use distinct random seeds. For each test instance and stochastic scenario, the same seed is assigned to all RGS variants at the corresponding repetition index, ensuring that the variants are evaluated using a common seed schedule rather than independently selected seed sets. However, stochastic values are generated sequentially during route construction, and different RGS variants may consume the random-number stream in different orders as their route trajectories diverge. An identical seed therefore does not necessarily produce identical customer-, arc-, or event-level realizations across variants. Accordingly, this protocol is treated as a matched-seed design that supports reproducibility and consistent evaluation conditions, rather than as a strict common-random-numbers design; no guaranteed variance reduction between methods is assumed.

In this study, $n_{\mathrm{test}}=6$ was fixed a priori as a moderate replication budget, providing 108 execution trajectories per policy and stochastic scenario while retaining all 18 test instances across the complete multi-scenario, multi-objective, and independently evolved policy design. This value is not claimed to guarantee Monte Carlo convergence. A smaller count would reduce evaluation cost but increase sensitivity to the particular sampled random-number streams, whereas a larger count would improve the precision of the estimated means and distributions, including the representation of less frequent extreme outcomes, without changing the evaluated policies. A dedicated convergence analysis across alternative values of $n_{\mathrm{test}}$ was not conducted. The reported distributional estimates are therefore conditional on the fixed evaluation budget, and additional independently generated execution sets could further improve their Monte Carlo precision.

All experiments were conducted on an MS Windows 11 workstation equipped with an AMD Ryzen Threadripper PRO 5995WX processor and 256 GB of DDR4-3200 memory (Advanced Micro Devices, Inc. (AMD), Santa Clara, CA, USA). The implementation used Jenetics 8.3.0 with its default execution settings; no additional parallelization or performance optimizations were applied. The reported online route-construction time of approximately 10 ms per 100-customer instance measures execution of the routing policy in the simulated environment, excluding offline GP training. It therefore isolates the downstream decision stage after the current state representation is available and does not include upstream sensing, communication, preprocessing, or data-fusion latency. To support reproducibility, the implementation is publicly available at: https://github.com/AlanDurdevic/stochastic-EVRP-GP (accessed on 8 September 2026).

**Figure 2:**
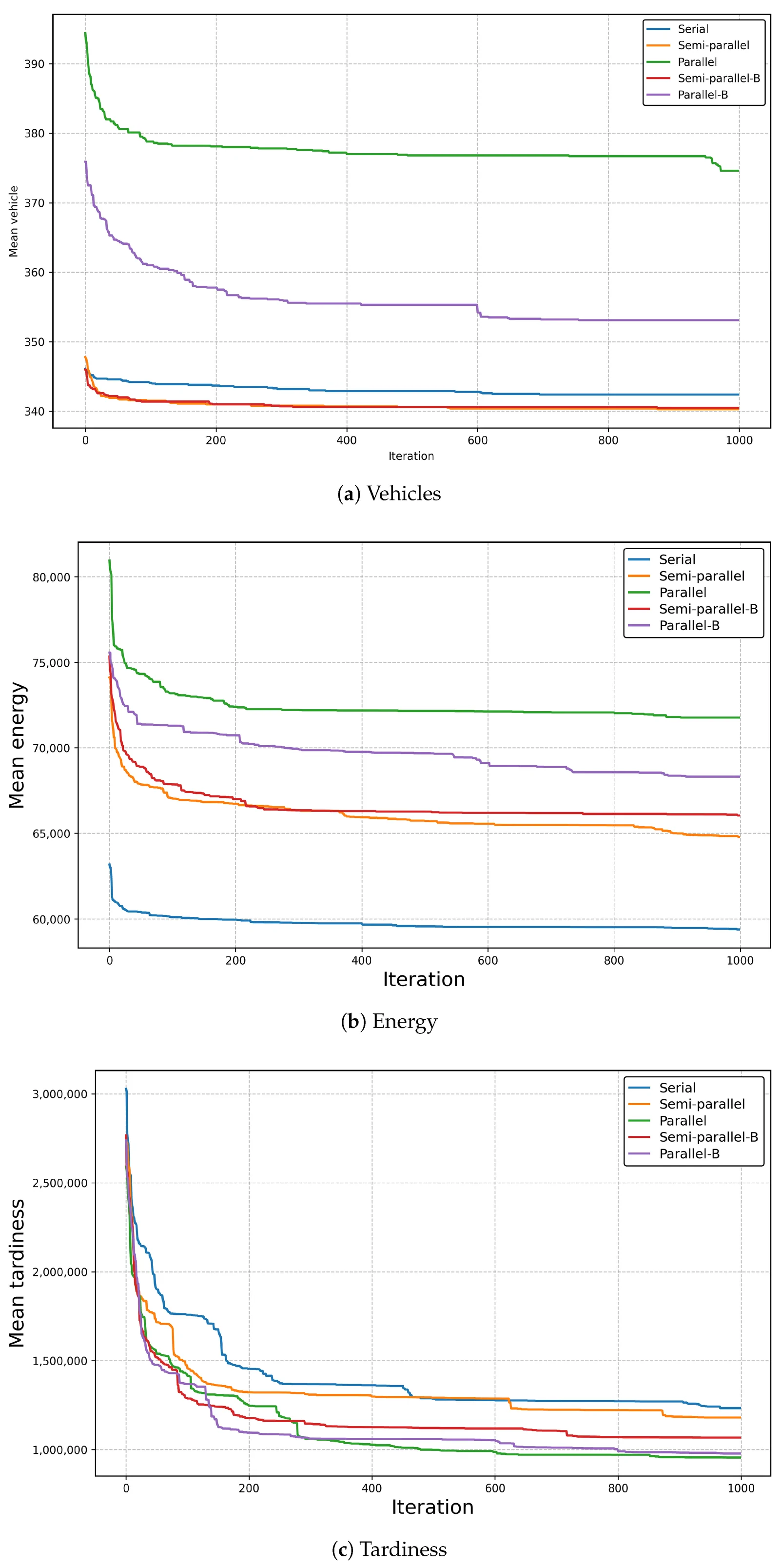
Mean best-so-far training objective value per generation across ten independent GP runs for the five RGS variants: (**a**) vehicle minimization, (**b**) energy minimization, and (**c**) tardiness minimization.

Figure 2 shows the mean best-so-far training objective value across the ten independent GP runs for each objective and RGS variant. All variants improved rapidly during the initial generations, followed by slower and less frequent improvements later in the evolutionary process. The final training order broadly agrees with the test results: the semi-parallel variants obtain the lowest fleet-size values, Serial obtains the lowest energy consumption, and the parallel variants obtain the lowest tardiness. The curves therefore indicate consistent optimization progress over the allocated evolutionary budget, although, as averages, they do not characterize variability between independent runs.

### 4.2. Results for Vehicle Minimization Objective

**Figure 3:**
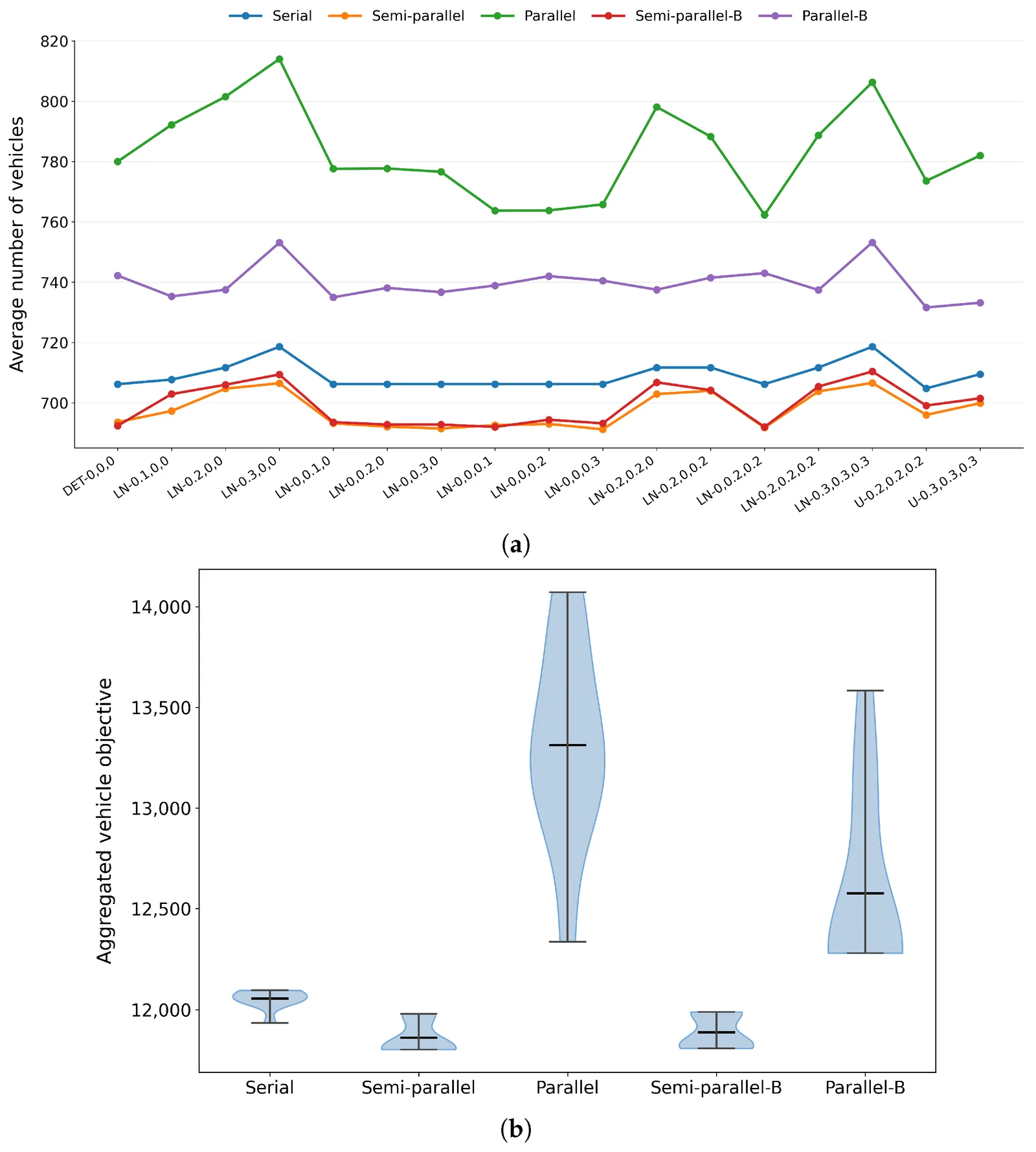
Results for the vehicle minimization objective. (**a**) Average number of vehicles for each RGS variant across all test scenarios. Scenario labels follow the format distribution-$CV_{d},CV_{s},CV_{v}$, where $CV_{d},CV_{s},CV_{v}$ denote variability in demand, service time, and vehicle speed, respectively. (**b**) Violin plots showing the distribution of vehicle counts for each RGS variant across all scenarios and runs.

Table 8 summarizes the results obtained when minimizing the number of vehicles. Figure 3 provides two complementary views of these results. The line plot (Figure 3a) shows the average number of vehicles across all test scenarios and serves as the primary basis for comparing the methods. Semi-parallel and Semi-parallel-B consistently achieve the lowest average values, with only minor differences between them across scenarios. Serial consistently performs slightly worse than these two variants, but remains clearly better than the Parallel-based variants. Among the latter, Parallel-B improves upon Parallel, although both remain substantially inferior to the Serial and Semi-parallel variants. This ordering is preserved across all scenarios, including the deterministic case (DET-0,0,0), indicating that stochasticity has little effect on the relative ranking of the RGS variants, although it does influence absolute performance levels.

The pooled distributions in Figure 3b show Semi-parallel and Semi-parallel-B concentrated at the lowest values, Serial slightly higher, and the Parallel variants shifted upward with broader distributions. This indicates that the Parallel-based variants have higher average objective values and broader pooled score distributions under stochastic conditions. For completeness, detailed violin plots for each individual test scenario are provided in Appendix A. Within the Parallel family, Parallel-B has lower averages and a narrower pooled distribution than Parallel. The difference between Semi-parallel and Semi-parallel-B is smaller: their mean values are similar, and their minimum values are frequently tied in Table 8.

Regarding the influence of stochasticity, demand variability has the most pronounced effect on fleet size. As the variability parameter of customer demand increases, the average number of vehicles also increases across all methods, with the effect being particularly strong for the Parallel-based variants, whose pooled distributions also broaden. In contrast, variability in service time has little to no impact on fleet size, with both average values and distributions remaining largely unchanged across scenarios. Variability in vehicle speed has a moderate effect. While the overall ranking of methods remains unchanged, speed variability leads to a slight increase in dispersion and, in some cases, small shifts in average performance.

### 4.3. Results for Energy Consumption Minimization Objective

**Figure 4:**
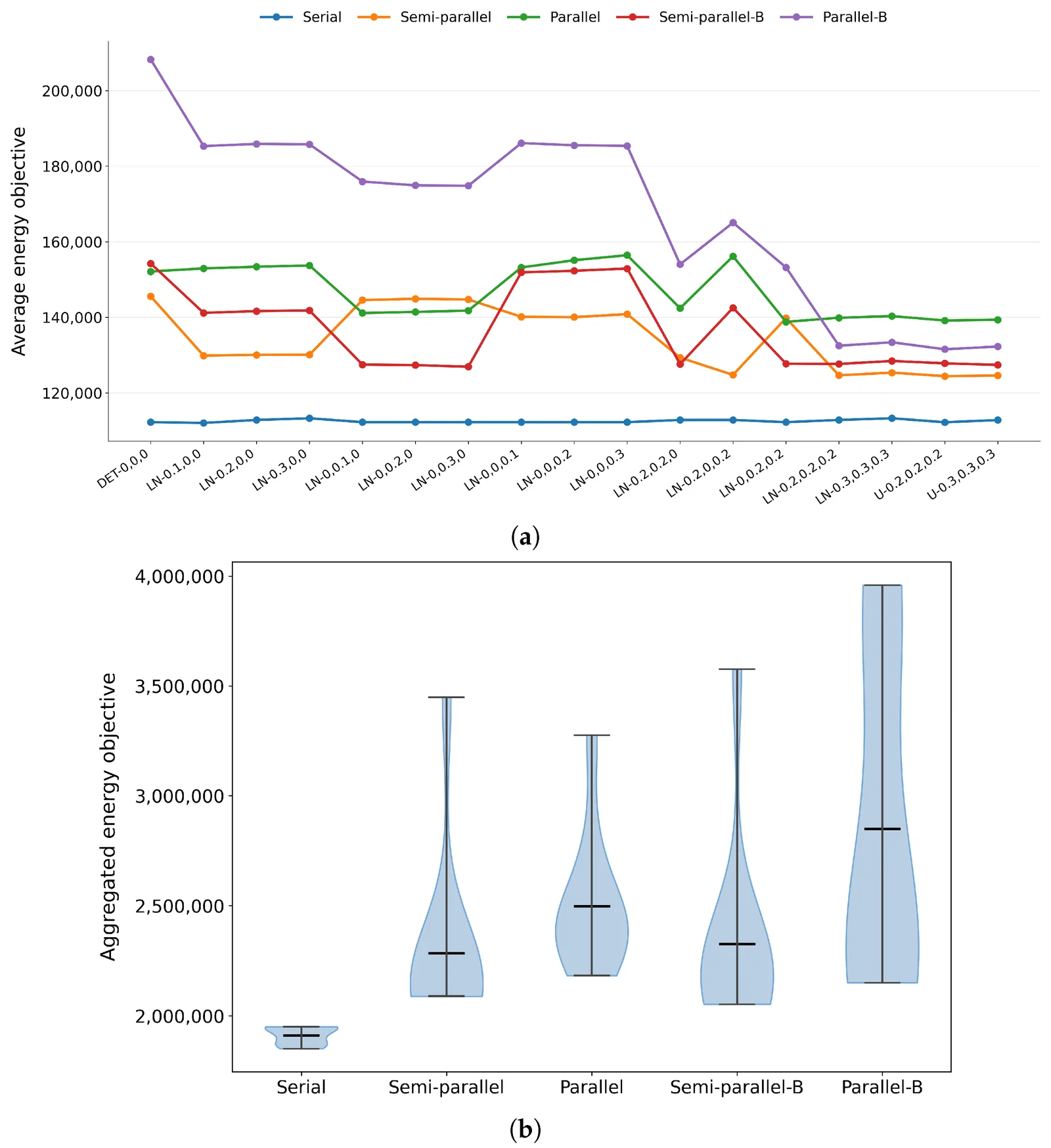
Results for the energy minimization objective. (**a**) Average energy consumption for each RGS variant across all test scenarios. Scenario labels follow the format distribution-$CV_{d},CV_{s},CV_{v}$, where $CV_{d},CV_{s},CV_{v}$ denote variability in demand, service time, and vehicle speed, respectively. (**b**) Violin plots showing the distribution of energy consumption for each RGS variant across all scenarios and runs.

Table 9 summarizes the results obtained when minimizing energy consumption, while Figure 4 shows the corresponding average values across test scenarios and distributions. Figure 4a shows a clear and consistent separation among the variants across scenarios: the Serial variant consistently achieves the lowest energy consumption in all scenarios. Notably, this behavior is also observed in the deterministic scenario (DET-0,0,0), showing that the energy advantage of the Serial variant is already present in the absence of stochastic perturbations and therefore cannot be attributed to uncertainty. Beyond this, the ordering of the remaining methods is less consistent. Semi-parallel and Semi-parallel-B generally outperform the Parallel-based variants, but their relative ranking varies across scenarios. Similarly, Parallel-B most often yields the highest energy consumption, although not in every instance. This indicates that, unlike the vehicle minimization objective, energy consumption is more sensitive to the interaction between routing variant and stochastic variability, leading to scenario-dependent performance differences among the non-serial variants.

The influence of stochasticity depends on the source of uncertainty. Demand variability has only a minor effect, producing small changes in average values without altering the overall structure of the results. In contrast, variability in service time has a more structural impact: while its effect on average energy consumption is moderate, it can alter the relative performance of closely competing methods. In particular, the ordering between Semi-parallel and Semi-parallel-B is occasionally reversed under service-time variability. Variability in vehicle speed has a weaker influence, introducing modest shifts in average performance and increased dispersion without changing the overall ranking. Additional insight is provided by the minimum values reported in Table 9. Although the B-variants often exhibit worse mean performance, Semi-parallel-B achieves a lower minimum than Semi-parallel across all scenarios, while Parallel-B does so in 15 of the 17 scenarios. These best-case improvements coexist with higher mean values and, particularly for Parallel-B, large standard deviations in several scenarios. The B-modification can therefore produce particularly energy-efficient individual policies, but this potential is not consistently realized across independently evolved PFs.

The pooled distributions in Figure 4b show Serial concentrated at low objective values with relatively limited dispersion. Semi-parallel and Semi-parallel-B exhibit a relatively compact spread, while the Parallel-based variants are shifted to higher values and display substantially greater dispersion. This is particularly evident for Parallel-B, which shows the widest spread and long upper tails, reflecting occasional highly inefficient solutions.

### 4.4. Results for Total Tardiness Minimization Objective

**Figure 5:**
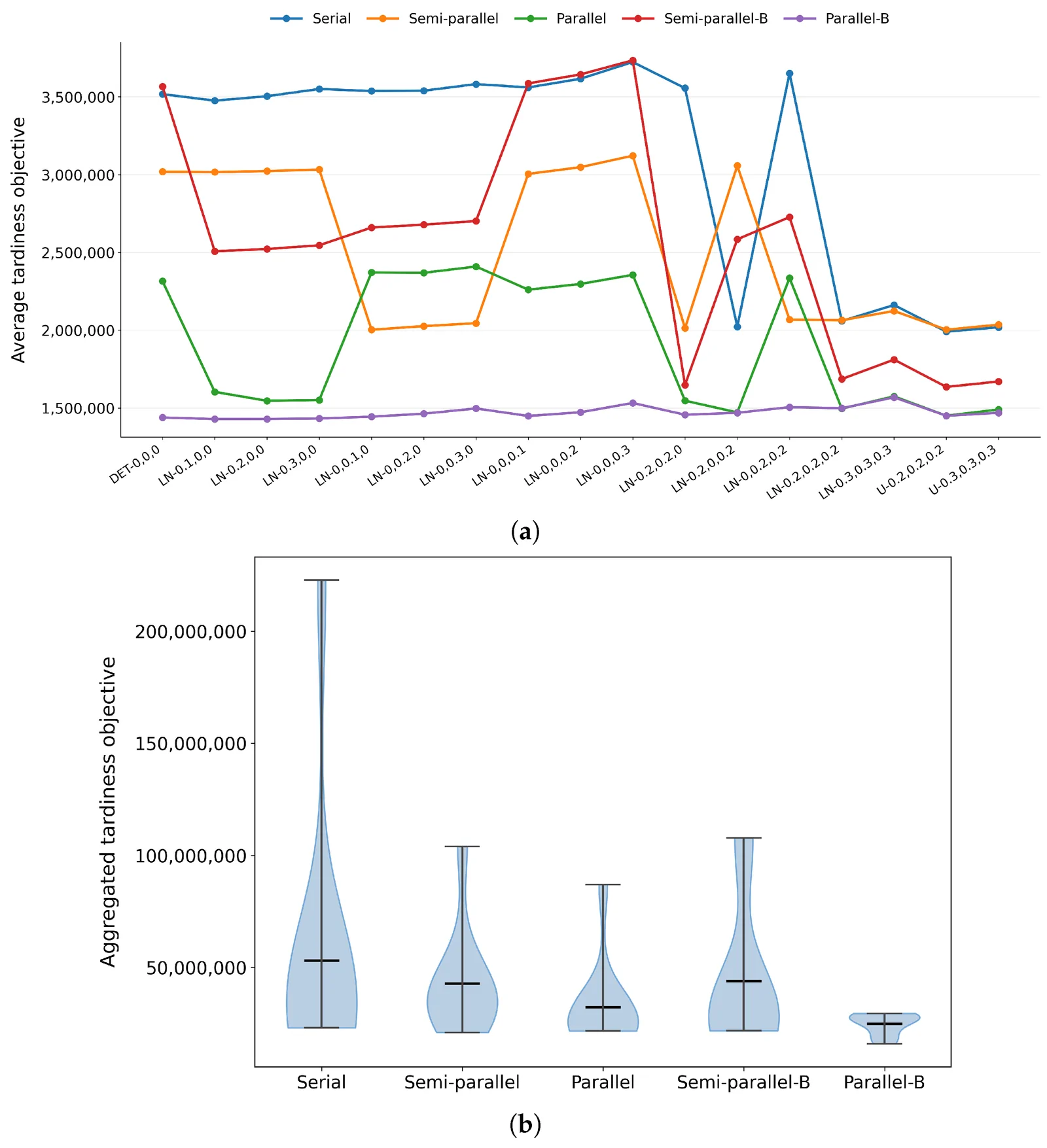
Results for the tardiness minimization objective. (**a**) Average tardiness for each RGS variant across all test scenarios. Scenario labels follow the format distribution-$CV_{d},CV_{s},CV_{v}$, where $CV_{d},CV_{s},CV_{v}$ denote variability in demand, service time, and vehicle speed, respectively. (**b**) Violin plots showing the distribution of tardiness for each RGS variant across all scenarios and runs.

Table 10 summarizes the results for the tardiness objective, while Figure 5 presents the corresponding averages and distributions. In contrast to the energy objective, the performance hierarchy is clearly inverted. As shown in Figure 5a, Parallel-B achieves the lowest average tardiness in 16 of the 17 scenarios, while Parallel is marginally best for LN-0.2,0.2,0.2. The remaining variants generally yield higher averages, although their relative ordering changes across scenarios. This indicates that increased parallelism can be beneficial for handling time-window constraints, although its effectiveness depends on the specific routing variant. This behavior is also observed in the deterministic scenario (DET-0,0,0), indicating that the advantage of Parallel-based variants is not driven by stochastic effects but reflects their ability to better handle time-window constraints.

Figure 5b shows Parallel-B with the lowest and narrowest pooled distribution. Parallel is also concentrated at relatively low values but has a longer upper tail. Serial is shifted to substantially higher values and has the broadest upper tail, while the semi-parallel variants lie between these groups.

The influence of stochasticity does not follow a simple or monotonic pattern. Variations in individual factors often have limited or inconsistent effects on their own, while combinations of stochastic elements lead to abrupt changes in performance. In particular, changes in demand alone have little impact on tardiness, whereas variability in service time and vehicle speed can trigger sharp increases for certain variants, most notably the Parallel variant. However, these effects are not cumulative in a predictable way: moderate combined uncertainty does not always increase tardiness and sometimes produces slightly lower averages.

### 4.5. Pairwise Comparison of Routing Variants

The descriptive results in Section 4.2, Section 4.3 and Section 4.4 are based on PF-level scores obtained by summing the objective values over the 108 evaluations performed for each scenario. For statistical analysis, the individual test-case outcomes were retained before this aggregation. For each objective, RGS variant, scenario, test instance, and stochastic realization, the ten PF outcomes were reduced to one median per RGS within each test condition and were therefore not treated as ten independent statistical observations. Each scenario–instance–realization combination therefore produced five corresponding RGS-specific medians and constituted one paired observation.

For each stochastic scenario, six distinct realizations were generated using independent random seeds and retained as separate observations, with pairing across RGS variants defined by the corresponding scenario, test instance, and repetition. Because the repetitions of the deterministic scenario produced identical outcomes, only one observation per instance was retained for that scenario. Consequently, each objective was analyzed using

16×18×6+18=1746

paired observations. Differences between the five RGS variants were evaluated using the Friedman test, followed by pairwise Wilcoxon signed-rank tests with Holm correction. These tests assess the consistency of differences across the evaluated test conditions, while variation among independently evolved PFs is characterized separately through the descriptive distributions in Section 4.2, Section 4.3 and Section 4.4.

The Friedman test identified statistically significant differences among the routing variants for all three objectives (vehicles: $\chi^{2}=3062.69$; energy: $\chi^{2}=4519.45$; tardiness: $\chi^{2}=3154.52$; all p<0.001), motivating the subsequent pairwise analysis. Table 11 reports the Holm-corrected Wilcoxon results together with the median paired difference $\tilde{\Delta }$ (row minus column). Negative values indicate that the row method tends to achieve lower objective values than the column method.

For vehicle minimization, all pairwise differences were statistically significant. Nevertheless, several comparisons had a median difference of zero, indicating that the differences were consistent across test cases but small in magnitude. Statistical significance for this objective should therefore be interpreted primarily in terms of consistency rather than practical effect size.

For energy minimization, all pairwise differences were also statistically significant. The median differences were generally larger than for vehicle minimization and agreed with the ordering observed in the aggregated results, with Serial achieving the lowest energy consumption and the Parallel-based variants generally performing worse.

For tardiness minimization, most pairwise differences were statistically significant. The comparison between Parallel and Parallel-B was the only nonsignificant result (p=0.289). The remaining comparisons confirm the general advantage of parallel route activation, although the magnitude of this advantage depends on the specific variant being compared.

### 4.6. Comparison with Simple Greedy Reference Heuristics

To provide additional context regarding the effectiveness of the proposed GP-based routing policies, we compare them against several simple constructive heuristics commonly used in routing settings:

- Nearest Neighbor (NN): selects the geographically closest feasible customer.
- Minimum Travel Energy (MTE): selects the customer with the lowest feasible travel-energy cost.
- Minimum Slack (MS): selects the customer with the smallest remaining time-window slack.
- Earliest Due Time (EDT): selects the customer with the earliest due time.

**Figure 6:**
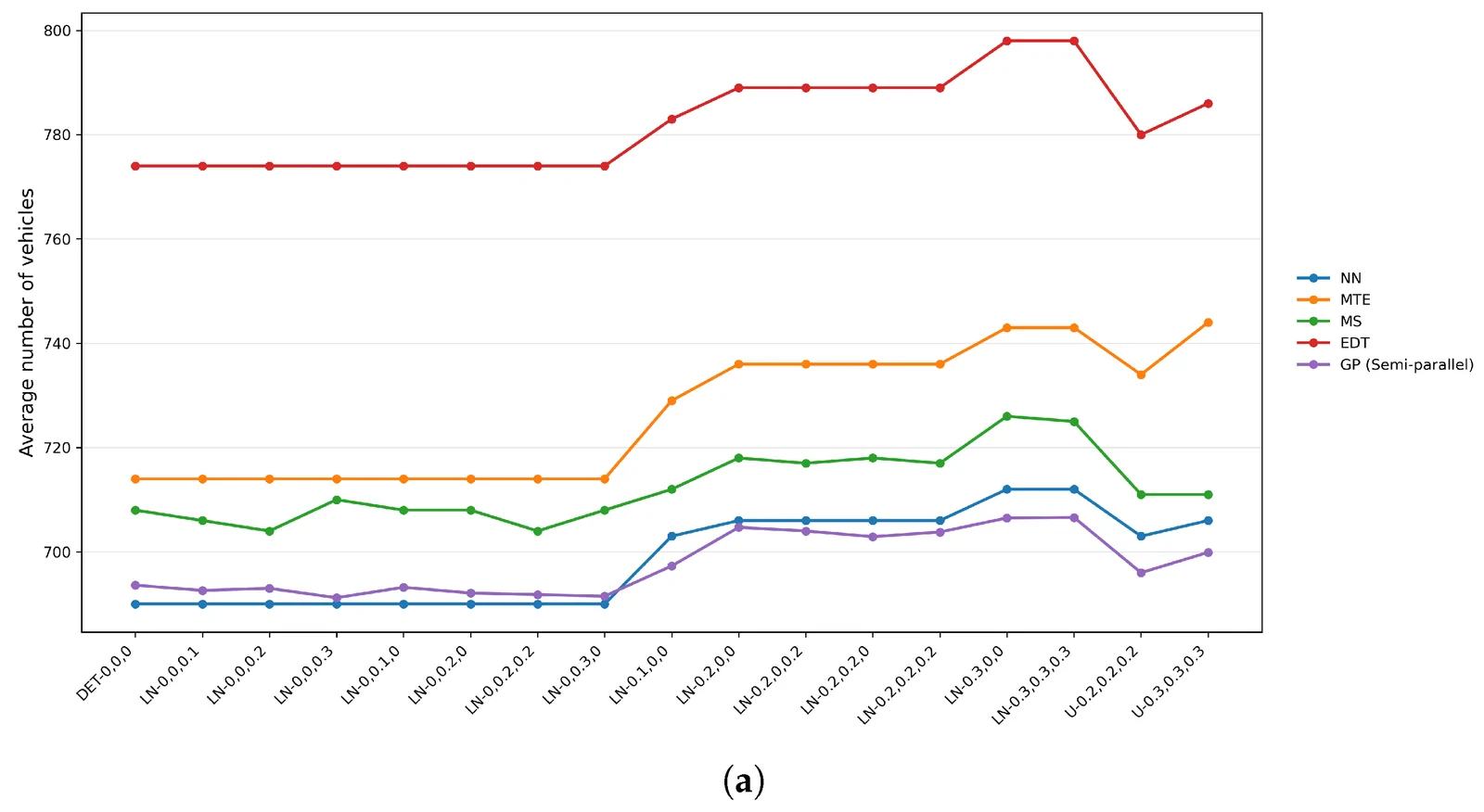

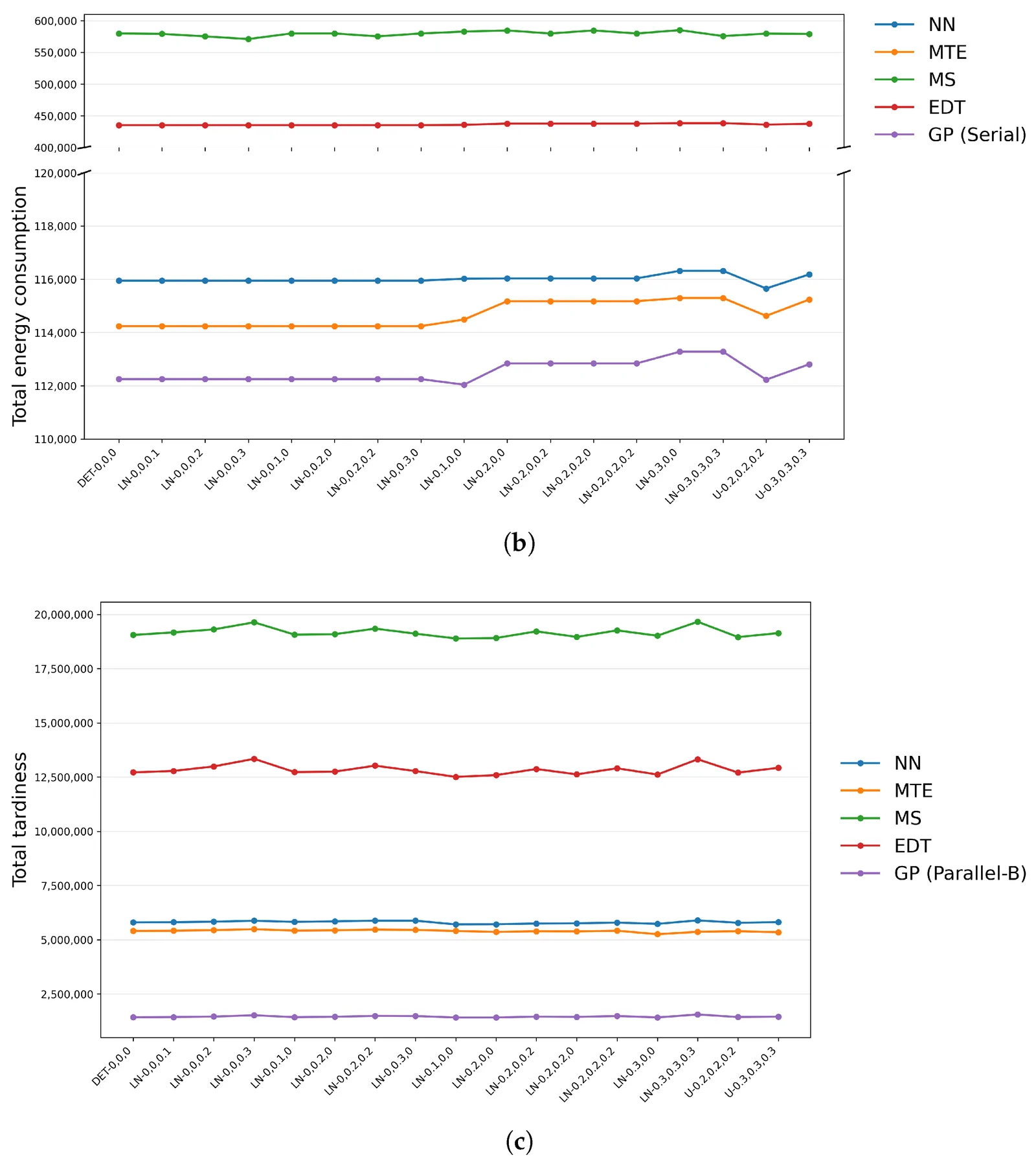
Comparison between simple untuned greedy reference heuristics using Serial route construction and the best-performing GP–RGS configuration for each objective across stochastic scenarios: (**a**) vehicle minimization, (**b**) energy minimization, and (**c**) tardiness minimization. Broken-axis visualization is used in the energy comparison to improve readability due to the large performance gap between methods.

All greedy heuristics were implemented within the same feasibility and route-construction framework as the proposed approach, replacing the GP-evolved priority function with the corresponding hand-designed customer-selection rule. The greedy heuristics use the Serial RGS as a common sequential route-construction setting and differ only in their customer-selection rules. They provide simple hand-designed reference points for the best-performing GP–RGS configuration identified for each objective. The observed differences therefore reflect the joint effect of the customer-selection policy and the route-generation mechanism. Since each reference heuristic represents a single deterministic policy, only aggregated objective values are reported. The results are shown in Figure 6.

The nearest-neighbor heuristic remains competitive for fleet-size minimization in deterministic and low-variability scenarios, occasionally achieving slightly lower values than the GP-based Semi-parallel policy. However, its fleet size increases more rapidly with demand variability than that of the GP-based Semi-parallel configuration.

For the energy objective, the GP-based Serial policy consistently achieves the lowest consumption across all tested scenarios. Although the minimum-travel-energy heuristic remains competitive, the remaining hand-designed heuristics perform substantially worse.

The largest performance differences are observed for tardiness, where the GP-based Parallel-B variant consistently outperforms all greedy references. This result reflects the joint contribution of the evolved priority function and the Parallel-B route-generation mechanism.

### 4.7. Structural and Semantic Analysis of Evolved Priority Functions

To examine the interpretability of the evolved routing policies, we analyzed the 150 best-of-run priority-function trees obtained across three objectives, five RGS variants, and ten independent GP runs. The analysis is descriptive and exploratory: it characterizes terminal usage, performance associations, and recurrent structural patterns in the observed trees.

Tree size was measured as the total number of function and terminal nodes, while tree depth was defined as the maximum number of edges from the root to a leaf. Table 12 reports medians and ranges calculated from the original, unsimplified best-of-run trees.

Vehicle-minimization trees were generally the most compact, particularly for Semi-parallel and Semi-parallel-B, both of which had a median size of three nodes and a median depth of one. Parallel was the main exception, producing substantially larger and more variable trees. Energy-minimization policies had intermediate complexity, whereas tardiness minimization generally produced the largest and deepest trees, especially for Serial and Semi-parallel. Parallel-B nevertheless produced relatively compact tardiness policies, with a median size of 22.5 nodes and a median depth of 9. The wide ranges observed in several configurations indicate substantial variation between independent runs and are consistent with occasional GP tree bloat.

Terminal usage was examined using relative occurrence frequencies. For terminal *t* in independently evolved tree *r*, let $c_{t,r}$ denote its number of occurrences and let $L_{r}$ denote the total number of terminal leaves in that tree. The relative usage frequency is defined as

(15) $$f_{t,r}=\frac{c_{t,r}}{L_{r}}.$$

This normalization prevents larger trees from receiving greater weight solely because they contain more nodes. Spearman correlations were used to characterize associations between $f_{t,r}$ and the corresponding objective values. Only correlations with p<0.1 are displayed, with those satisfying p<0.05 marked separately. Given the limited number of independently evolved trees per configuration, these correlations are interpreted as exploratory associations rather than estimates of a causal performance contribution.

**Figure 7:**
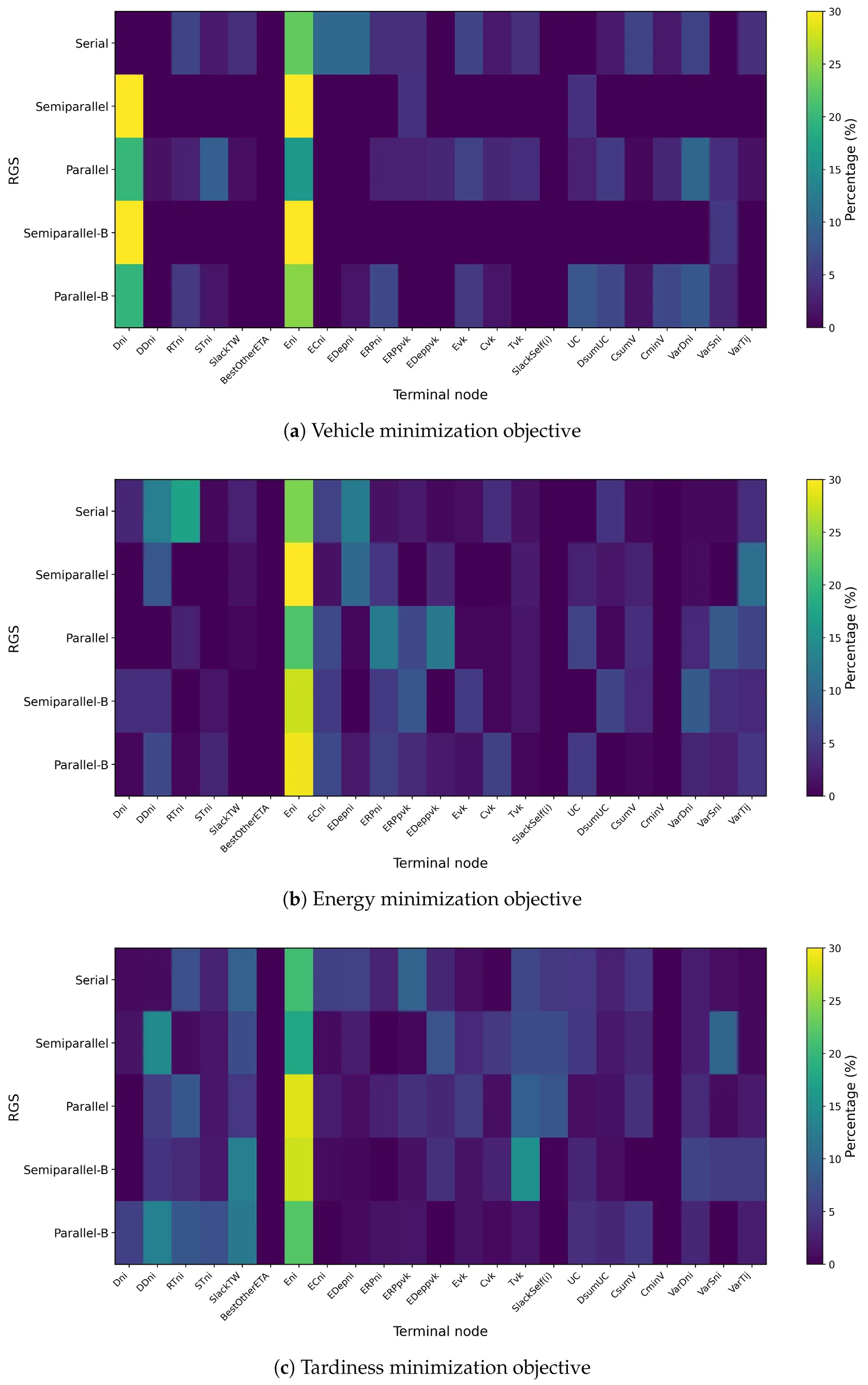
Relative frequencies of terminal nodes used in the evolved priority functions for each routing variant and optimization objective.

**Figure 8:**
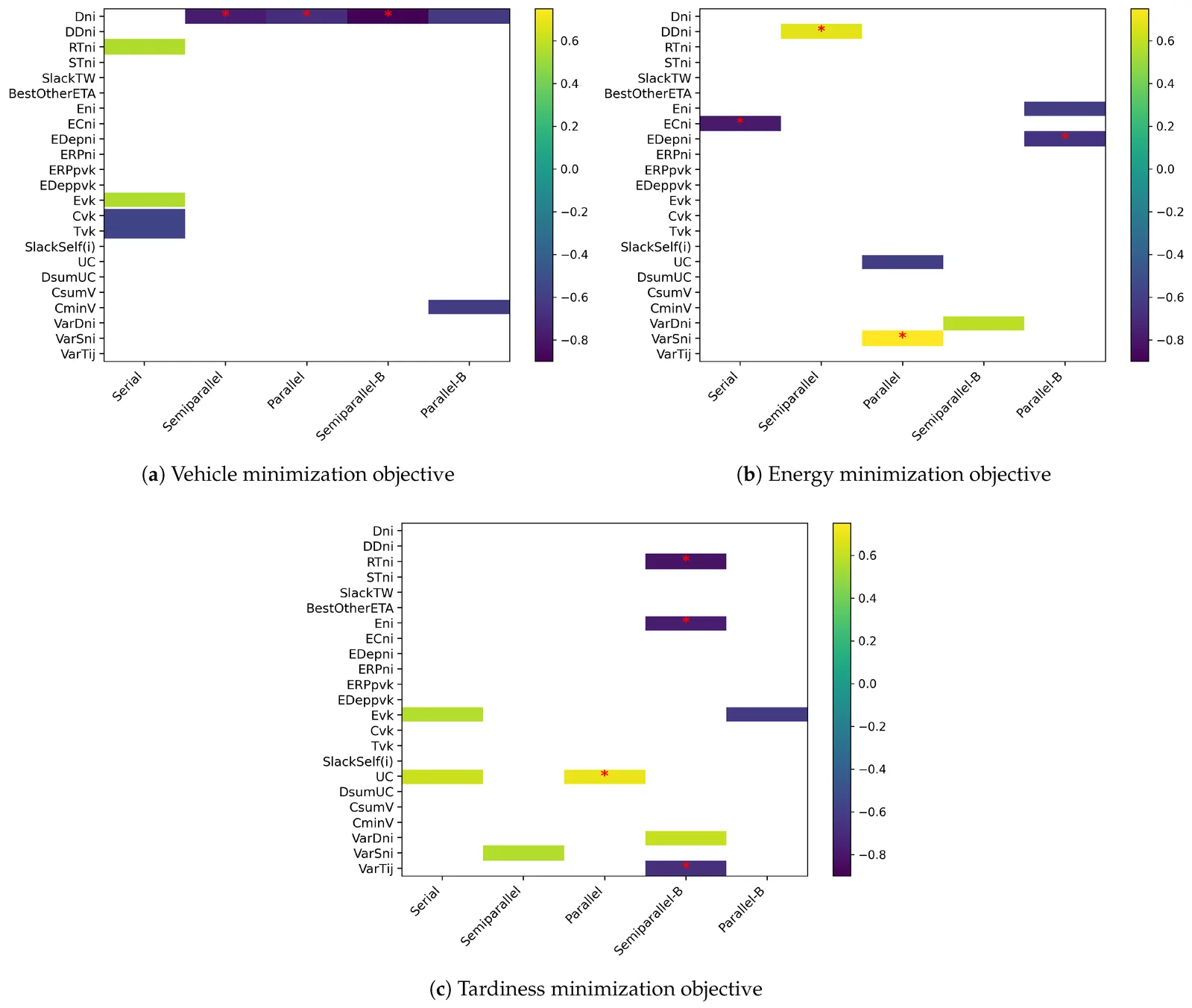
Correlation between terminal node usage frequency and objective values for each routing variant and optimization objective. The values represent Spearman rank correlation coefficients. Only correlations with p<0.1 are shown. Statistically stronger correlations (p<0.05) are marked with a red asterisk.

For vehicle minimization (Figure 7a, terminal usage in Semi-parallel and Semi-parallel-B was strongly concentrated on $D_{ni}$ and $E_{ni}$. The Parallel variants used the same terminals within a more distributed representation, while Serial exhibited the most dispersed profile. More frequent use of $D_{ni}$ correlated negatively with fleet size across the semi-parallel and parallel variants (Figure 8a). Despite the high frequency of $E_{ni}$, none of its correlations with fleet size met the p<0.1 reporting threshold. Serial instead exhibited stronger associations for timing- and vehicle-state terminals such as $RT_{ni}$ and $E_{vk}$.

$E_{ni}$ was the most frequently used terminal across all energy-minimization variants, particularly the semi-parallel variants (Figure 7b). Serial also made notable use of $RT_{ni}$, the due-time terminal $DD_{ni}$, and $EDep_{ni}$, whereas Parallel used a broader terminal set that included the stochastic descriptors $Var_{Dni}$, $Var_{Tij}$, and $Var_{Sni}$. When terminal frequencies were correlated with energy consumption, the terminals meeting the reporting threshold differed by RGS (Figure 8b). $EC_{ni}$ correlated negatively with energy consumption in Serial, $DD_{ni}$ correlated positively in both semi-parallel variants, and $EDep_{ni}$ correlated negatively in Parallel-B. Parallel showed correlations for several terminals, most notably $Var_{Sni}$.

For tardiness minimization (Figure 7c), $E_{ni}$ remained the most frequent terminal, while Parallel and Parallel-B used a broader set of timing-, vehicle-state-, and system-level descriptors. When terminal frequencies were correlated with total tardiness, the terminals meeting the reporting threshold again differed by RGS (Figure 8c). Within Parallel, UC showed the strongest positive correlation with total tardiness. In Parallel-B, several terminals met the threshold, including $RT_{ni}$, $E_{ni}$, and $Var_{Tij}$, whereas only isolated terminal correlations met the threshold in the remaining variants.

Terminal frequencies do not reveal whether decision signals are combined into recurrent local structures. We therefore examined the predefined semantic patterns listed in Table 13. A pattern was considered local when its participating terminals were each located at most two edges from their lowest common ancestor. This threshold captures terminals combined within a small shared subtree while avoiding the interpretation of distant occurrences as a structural pattern. A direct pattern, defined as sibling leaves of the same function node, represents the most restrictive form of local association. For semantic pattern *M*, empirical support is defined as

(16) $$S_{M}=\sum_{r=1}^{n_{\mathrm{runs}}}1\;\left(M\in T_{r}\right),$$

where $n_{runs}=10$, $T_{r}$ is the priority-function tree obtained in independent run *r* and 1(·) denotes the indicator function. Specifically, $1(M\in T_{r})$ equals one if tree $T_{r}$ contains at least one occurrence satisfying the predefined structural condition for pattern *M*, and zero otherwise. Each tree therefore contributes at most once to the support of a given pattern, regardless of how many times that pattern occurs within the tree. Accordingly, $S_{M}$ ranges from 0 to $n_{\mathrm{runs}}$, and $S_{M}/n_{\mathrm{runs}}$ is interpreted as empirical recurrence across the independently evolved policies.

For vehicle minimization, the clearest recurrent pattern combined customer demand $D_{ni}$ with the energy required to visit the customer $E_{ni}$ (Table 13). The pattern was absent from Serial but occurred locally in at least half of the trees for every other variant. It was also direct in 6/10 trees for both semi-parallel variants. In Parallel-B, by contrast, it occurred locally in 6/10 trees but directly in only 2/10, indicating greater syntactic variation. The observed support shows that $D_{ni}$ and $E_{ni}$ co-occurred locally in multiple independently evolved trees, particularly in the semi-parallel policies. This establishes structural recurrence within the analyzed trees but does not isolate the contribution of the pattern to performance.

For energy minimization, $E_{ni}$ occurred in all ten trees for every RGS variant, but no equally recurrent local structure was observed. No specific pair involving $E_{ni}$ reached a support of 6/10. The highest support for an exact pair was 4/10 for $E_{ni}$–$EC_{ni}$ in Parallel-B, while the broader combination of visit energy with depot- or charging-related energy reached 6/10 only in Semi-parallel. Thus, independently evolved policies consistently used $E_{ni}$ but combined it with heterogeneous secondary signals.

Tardiness policies exhibited the strongest consistency at the semantic-group level. Energy and temporal or urgency-related terminals occurred locally in 6/10 to 9/10 trees across all RGS variants, but the specific temporal partner varied. Only $E_{ni}$–Slack_TW in Semi-parallel-B reached a support of 6/10 as an exact pair. Energy–stochastic combinations also reached 6/10 in Semi-parallel-B but were less frequent elsewhere. The observed supports indicate repeated energy–time co-occurrence at the semantic-group level despite variation in the specific terminals and GP expressions.

Across the best-performing configurations for the three objectives, descriptors related to demand, energy, timing, and fleet state were more recurrent than explicit variability descriptors. Operational-state descriptors appeared more consistently in the analyzed trees than explicit distributional summaries, although the observed frequencies remained objective- and RGS-dependent.

## 5. Discussion

### 5.1. Vehicle Minimization

The results broadly follow the main conclusions reported in [29], where Serial and Semi-parallel outperform Parallel in terms of fleet size. Under the stochastic conditions considered here, both semi-parallel variants achieve lower average fleet sizes than Serial across all evaluated scenarios, indicating a more consistent advantage for configurations with limited parallel route construction. One possible interpretation is that the semi-parallel schemes provide access to multiple active vehicles while avoiding the continuous replacement of completed routes used by the Parallel schemes. However, route fragmentation and capacity-utilization efficiency were not measured directly, so these mechanisms cannot be established from the present results. Moreover, because the semi-parallel and parallel families are initialized with LB active vehicles, their comparison with Serial reflects the combined effects of route parallelism and LB-based initialization rather than the isolated effect of either design choice. The B-modification changes capacity allocation by selecting the vehicle with the largest remaining capacity among the *K* earliest available vehicles, but its effect within the semi-parallel family is small and scenario-dependent; Semi-parallel-B does not consistently outperform Semi-parallel. Among the considered uncertainty sources, demand variability has the strongest influence on fleet size, with average fleet size increasing as demand variability rises. Service-time variability has little effect, whereas travel-time variability primarily affects dispersion without changing the general ordering of the routing variants. Although all pairwise differences for vehicle minimization are statistically significant, several have a median paired difference of zero. These results therefore indicate consistent but practically small differences among some variants.

The structural and semantic analysis identifies policy characteristics associated with the effective semi-parallel configurations. Semi-parallel and Semi-parallel-B evolved particularly compact policies, with a median tree size of three nodes and a median depth of one. In both variants, $D_{ni}$ and $E_{ni}$ appeared as direct sibling terminals in 6/10 trees, showing repeated joint use of customer demand and the energy required to visit the customer. More frequent use of $D_{ni}$ also correlated with smaller fleet sizes across the semi-parallel and parallel variants. The local $D_{ni}$–$E_{ni}$ pattern was absent from Serial, whose displayed correlations instead involved timing- and vehicle-state descriptors. Parallel and Parallel-B also contained the pattern, but generally within larger or more varied expressions. These observations show that compact rules containing both demand and visit-energy terminals recurred in the semi-parallel configurations. The support counts do not, however, establish that this structure caused their fleet-size performance. No recurrent structural evidence was observed for a prominent use of explicit variability descriptors in vehicle minimization.

### 5.2. Energy Minimization

For the energy minimization objective, the relative behavior of routing variants changes substantially. Consistent with [29], the Serial variant achieves the best overall performance. Energy minimization favors sequential route construction, and Serial retains the lowest average consumption as travel-time variability increases. The Parallel-based variants produce higher averages and broader pooled distributions, particularly Parallel-B. The semi-parallel variants occupy an intermediate position in both average energy and dispersion.

The structural analysis does not indicate a simple relationship between policy complexity and energy performance: Serial and Semi-parallel had similar median tree sizes and depths, while all variants occasionally produced substantially larger trees. At the semantic level, $E_{ni}$ occurred in every independently evolved energy-minimization policy, showing its consistent inclusion across all RGS variants. However, no specific local pair involving $E_{ni}$ recurred in at least 6/10 trees, and the broader combination of visit energy with depot- or charging-related energy reached this support only in Semi-parallel. Thus, the policies consistently included the immediate energy required to visit a customer but combined it with heterogeneous secondary signals. In Serial, more frequent use of $EC_{ni}$ correlated with lower energy consumption. Since $EC_{ni}$ measures the energy required to travel from a candidate customer to the centroid of the remaining unserved customers, it incorporates information about the candidate’s position relative to the remaining service set. Parallel used a broader set of energy and stochastic descriptors but achieved poorer energy results, indicating that greater terminal diversity alone does not translate into better energy performance.

### 5.3. Tardiness Minimization

Unlike energy minimization, tardiness minimization favors early and distributed route activation. Consistent with [29], the Parallel-based variants achieved the best overall performance, as distributing customers among routes limits the accumulation of delays along individual routes. Serial construction activates additional vehicles only when needed and therefore provides less flexibility once delays accumulate. Parallel-B had lower scenario averages and a narrower pooled distribution than Parallel, but their paired difference was not statistically significant. The larger differences between the parallel and less-parallel variants suggest that early route activation is the more consequential design choice. The influence of stochasticity was less structured, with particular uncertainty configurations producing abrupt performance changes and occasional reductions in average tardiness. These reductions primarily reflected a contraction of the upper tail rather than uniform improvement across evolved policies. For example, for Parallel, mean tardiness decreased from approximately $2.32\times 10^{6}$ in the deterministic scenario to $1.60\times 10^{6}$ under LN-0.1,0,0, while the standard deviation across PF-level scores decreased from $2.30\times 10^{6}$ to $0.48\times 10^{6}$. Together with the corresponding distributions in Figure 5, this indicates that the change primarily reflects a contraction of high-tardiness outcomes rather than uniform improvement across evolved policies. Stochastic perturbations can alter the decisions made during adaptive route construction and occasionally avoid sequences that produce extreme delays, whose contribution is amplified by the soft time-window objective. Because comparable non-monotonic changes also occur for other RGS variants, parallel route activation alone does not explain this behavior. Such reductions are therefore scenario-dependent and should not be interpreted as evidence that uncertainty is generally beneficial.

Tardiness minimization produced the largest and deepest policies overall, consistent with the need to combine energy feasibility with time-window and vehicle-state information. Despite considerable syntactic variation, the support analysis showed repeated local co-occurrence of energy-related and temporal or urgency-related terminals: local combinations of energy-related and temporal or urgency-related terminals occurred in 6/10 to 9/10 trees across all RGS variants. The concrete temporal partner varied, showing recurrence at the semantic-group level without a common expression-level structure. Parallel showed both one of the highest supports for this pattern and the best tardiness performance; however, similarly frequent energy–time combinations also occurred in less successful variants, showing that the motif alone does not determine performance. The RGS controls how these signals are translated into route-construction decisions. Notably, Parallel-B achieved performance comparable to Parallel using substantially more compact policies, indicating that large trees are not required for effective tardiness minimization. Although UC had the strongest displayed correlation in Parallel, the direction was positive: more frequent use coincided with greater tardiness. This identifies UC as a performance-related descriptor but does not imply that its use improves coordination. Energy–stochastic patterns were uncommon in the best-performing parallel variants, whereas realized timing and system-state descriptors appeared more frequently.

### 5.4. Overall Observations

Taken together, the results show that no single RGS variant is optimal across all objectives. Vehicle minimization favors limited parallelism, energy minimization favors sequential route construction, and tardiness minimization benefits from early workload distribution across multiple vehicles. Across the evaluated scenarios and independently sampled realizations, stochasticity affected absolute performance and dispersion more strongly than the identity of the best-performing RGS family.

The structural analysis likewise showed that observed policy organization varied across objectives and RGS variants and reflected the combination of the evolved priority function and its route-generation mechanism. Policy size alone did not predict performance. Across the best-performing configurations, descriptors related to demand, energy, timing, and fleet state were more recurrent than explicit variability descriptors. From a sensing perspective, this pattern shows that the evolved policies repeatedly incorporated objective-relevant operational-state variables, whereas explicit distributional information was used less consistently and remained objective- and policy-dependent.

The structural results identify recurrent representation patterns but do not establish statistical overrepresentation or the functional necessity of individual terminals. Testing motif enrichment would require a structural null model that accounts for tree topology, marginal terminal frequencies, function arities, and structural biases introduced by GP initialization, variation, and selection. Testing terminal necessity would instead require controlled re-evolution under a modified primitive set, allowing GP to construct compensating expressions from the remaining terminals. These analyses address distinct questions beyond the descriptive scope of the present study.

From an operational perspective, the three objectives represent different fleet-management priorities: resource use, energy efficiency, and service quality. The GP–RGS configuration should therefore be selected according to the relevant objective rather than expecting one configuration to perform best across all criteria. Once evolved, the priority functions construct routes in approximately 10 ms per 100-customer instance, allowing updated operational states to be translated into routing actions with low online computational overhead. This supports the use of the framework as a downstream decision layer in sensing-enabled fleet-management systems.

More broadly, the proposed policy-learning framework complements mathematical-optimization approaches based on decomposition, robust optimization, and stochastic programming [44,45,46]. Whereas such methods typically solve a specified problem instance and produce an instance-specific solution, the proposed framework incurs its main computational cost during offline evolution across training instances and subsequently applies the resulting priority functions to unseen instances as operational information is revealed.

The two paradigms therefore allocate computational effort differently. In the proposed framework, the offline evolutionary cost is shared across subsequent applications of the learned policy, and its contribution to the amortized cost per application decreases as the policy is reused. An instance-specific optimization method instead incurs a new solve cost for each supplied instance, but can devote that computation to the complete formulation of the particular problem. Reusable policy learning consequently emphasizes low online response latency and repeated application, whereas instance-specific optimization emphasizes solution construction for the currently supplied instance. The reported online runtime excludes GP evolution and therefore does not establish lower total computational complexity or general asymptotic superiority over mathematical-optimization approaches. Meaningful comparisons of solution quality, total computational cost, or scalability would require a common problem formulation, objective set, information structure, stopping criterion, evaluation protocol, and computational environment.

### 5.5. Limitations and Future Work

The conclusions are limited to the fixed 100-customer benchmark scale, independently generated parametric uncertainty, and predefined information-revelation events. The decision-level voting count was fixed at $n_{\mathrm{vote}}=5$. Because this count affects action selection during fitness evaluation, examining alternative values requires independent policy evolution under each voting budget. The test protocol likewise used $n_{\mathrm{test}}=6$ independently seeded executions per held-out instance as a fixed Monte Carlo evaluation budget. Increasing this count would improve the precision of the estimated performance distributions without changing the evaluated policies. No dedicated sensitivity analysis of $n_{\mathrm{vote}}$ or convergence analysis of $n_{\mathrm{test}}$ was conducted; the reported comparisons are therefore conditional on these fixed budgets.

Future work will examine alternative voting budgets through independent policy evolution and evaluate Monte Carlo convergence using larger, repeatedly generated test sets. Controlled primitive-set ablation and topology-preserving null-model analyses could distinguish empirical terminal recurrence from functional dependence and statistical motif enrichment. Further research will consider richer and correlated uncertainty models, continuous or asynchronous operational updates, risk-adjusted vehicle-initialization rules, broader problem scales, and comparisons with alternative online decision methods under aligned information and computational assumptions.

## 6. Conclusions

This study extended a GP-based route-generation framework to the stochastic electric vehicle routing problem with time windows. Controlled stochastic realizations of customer demand, service time, and travel time provide a technology-agnostic representation of progressively revealed operational information. The framework combines stochastic and global-state priority-function descriptors with two capacity-aware route-generation variants, Semi-parallel-B and Parallel-B, producing routing policies that can be reused on unseen instances and adapt route construction without repeatedly solving an optimization problem.

The results show that the most suitable route-generation scheme depends on the operational objective. Semi-parallel variants achieved the lowest fleet-size values, Serial consistently achieved the lowest energy consumption, and Parallel-based variants achieved the lowest tardiness. Across the evaluated uncertainty scenarios, stochasticity affected the magnitude and dispersion of performance more strongly than the identity of the best-performing RGS family. The structural analysis identified objective- and RGS-dependent policy organization, with operational-state descriptors recurring more consistently than explicit variability descriptors. The GP-based configurations also remained competitive with simple constructive heuristics and achieved their clearest advantage for tardiness minimization.

Within a sensing-enabled fleet-management system, the proposed framework provides a downstream decision layer for converting operational-state updates into adaptive routing actions. Applying an already evolved policy required approximately 10 ms per 100-customer instance on average, demonstrating low online computational cost at the studied scale, although this measurement excludes offline GP evolution. Overall, the results support reusable GP-based policies as a promising approach to adaptive route construction under simultaneous operational uncertainty.

## Figures and Tables

**Table 1:** Notation used in the EVRPTW formulation.

Symbol	Definition
$N_{c}$	Set of customer nodes.
$N_{s}$	Set of charging-station nodes.
0	Depot node.
$N=\left\{0\right\}\cup N_{c}\cup N_{s}$	Set of all nodes.
*L*	Set of links connecting the nodes.
$dist_{ij}$	Distance between nodes *i* and *j*.
$d_{i}$	Nominal demand of customer *i*.
$s_{i}$	Nominal service time of customer *i*.
$\left[a_{i},b_{i}\right]$	Service time window associated with customer *i*.
*C*	Vehicle cargo capacity.
*Q*	Vehicle battery capacity.
*v*	Nominal vehicle speed.
*r*	Energy-consumption rate.
*g*	Charging rate.
$A_{i}$	Arrival time at customer *i*.
$F_{i}$	Completion time of service at customer *i*.
$T_{i}$	Tardiness at customer *i*, defined as $T_{i}=\max\left(F_{i}-b_{i},0\right)$.

**Table 2:** Variant-specific vehicle management rules in the unified RGS framework.

Variant	Initialization of *V*	Vehicle Selection	Update After Route Completion
Serial	∅	activate a new vehicle	no change
Semi-parallel	{1,2,…,LB}	if V=∅, activate a new vehicle; otherwise select the earliest available vehicle from *V*	V←V∖{vehicle}
Parallel	{1,2,…,LB}	select the earliest available vehicle from *V*	V←(V∖{vehicle})∪{newvehicle}
Semi-parallel-B	{1,2,…,LB}	if V=∅, activate a new vehicle; otherwise select the vehicle with the largest remaining capacity among the *K* earliest available vehicles in *V*	V←V∖{vehicle}
Parallel-B	{1,2,…,LB}	select the vehicle with the largest remaining capacity among the *K* earliest available vehicles in *V*	V←(V∖{vehicle})∪{newvehicle}

*Note: V* denotes the set of currently active vehicles, and vehicle denotes the vehicle whose route has just been completed.

**Table 3:** Configuration of the GP algorithm.

Parameter	Value
Initialization	Full
Population size	200
Number of generations	1000
Initial tree depth	5
Maximum tree depth	$2^{8}-1$
Elitism	1
Survivor selector	Tournament selection (size 3)
Offspring selector	Tournament selection (size 3)
Offspring fraction	0.05
Crossover operator	Subtree crossover
Mutation operator	Subtree mutation
Mutation rate	0.2

**Table 4:** Terminal nodes used in the GP representation.

Terminal	Objective	Description
$E_{ni}$	all	Energy required to visit customer *i*.
$D_{ni}$	all	Demand of customer *i*.
$DD_{ni}$	all	Due time of customer *i*.
$ST_{ni}$	all	Service time of customer *i*.
$RT_{ni}$	all	Ready time of customer *i*.
$E_{vk}$	all	Remaining battery charge of vehicle *k*.
$C_{vk}$	all	Remaining cargo capacity of vehicle *k*.
$T_{vk}$	all	Current route time of vehicle *k*.
$EC_{ni}$	all	Energy required to travel from node *i* to the centroid of unserved customers.
$ERP_{ni}$	all	Energy required to travel from node *i* to the nearest charging station.
$EDep_{ni}$	all	Energy required to travel from node *i* to the depot.
$ERP_{pvk}$	all	Energy required to travel from the current position *p* of vehicle *k* to the nearest charging station.
$EDep_{pvk}$	all	Energy required to travel from the current position *p* of vehicle *k* to the depot.
Var_Dni	all	Demand variability parameter for customer *i*.
Var_Tij	all	Travel-time variability parameter for customer *i*.
Var_Sni	all	Service time variability parameter for customer *i*.
Slack_TW	all	Remaining time window slack for link (i,j).
UC	all	Number of unserved customers.
DsumUC	all	Total demand of all unserved customers.
CsumV	all	Total remaining capacity of all active vehicles.
$BestOtherETA_{i}$	all	Minimum estimated arrival time to customer *i* among the *K* earliest available vehicles excluding the currently considered vehicle.
CminV	vehicle	Minimum remaining capacity among all active vehicles.
SlackSelf(i)	tardiness	Available slack of the currently considered vehicle for customer *i*, computed as the difference between the customer’s due time and the estimated arrival time of the vehicle.

**Table 5:** Function nodes used in the GP representation.

Function Nodes						
add	sub	mul	div	max	min	
neg	pow2	sqr	exp	log	max0	min0

*Note:* max0(x)=max(x,0) and min0(x)=min(x,0).

**Table 6:** Main factors affecting online GP–RGS execution cost.

Quantity	Role	Cost Effect
*n*	Number of customers	Up to quadratic if D=O(n)
*m*	Number of charging stations	Linear: O(mD)
*D*	Route-extension decisions	Linear in decision-dependent terms
$n_{\mathrm{vote}}$	Evaluations per customer and decision	Linear in priority evaluation
|T|	Priority-function tree size	Linear in priority evaluation
*K*	Vehicles inspected by B-variants	Linear: O(KD)

**Table 7:** Evaluation scenarios grouped by uncertainty configuration.

Scenario Group	Scenarios
Deterministic baseline	DET-0,0,0
Demand variability only	LN-0.1,0,0, LN-0.2,0,0, LN-0.3,0,0
Service-time variability only	LN-0,0.1,0, LN-0,0.2,0, LN-0,0.3,0
Vehicle-speed variability only	LN-0,0,0.1, LN-0,0,0.2, LN-0,0,0.3
Pairwise lognormal variability	LN-0.2,0.2,0, LN-0.2,0,0.2, LN-0,0.2,0.2
Joint lognormal variability	LN-0.2,0.2,0.2, LN-0.3,0.3,0.3
Joint uniform variability	U-0.2,0.2,0.2, U-0.3,0.3,0.3

**Table 8:** Results for the vehicle minimization objective across all test scenarios. Each PF score is aggregated over 108 evaluations. Each cell reports the mean ± standard deviation across the ten independently evolved PFs on the first line and the minimum obtained value on the second line. Best mean results are shown in bold.

Test Type	Serial	Semi-Parallel	Parallel	Semi-Parallel-B	Parallel-B
DET-0,0,0	706.2±5.7 *min*: 690	693.6±5.8 *min*: 690	780.0±51.0 *min*: 714	692.4±3.1 *min*: 690	742.2±54.1 *min*: 708
LN-0.1,0,0	707.7±2.0 *min*: 706	697.3±8.8 *min*: 691	792.2±50.2 *min*: 725	702.9±8.0 *min*: 694	735.3±16.4 *min*: 721
LN-0.2,0,0	711.7±2.0 *min*: 710	704.7±5.9 *min*: 700	801.5±47.2 *min*: 728	706.0±5.2 *min*: 701	737.5±18.8 *min*: 727
LN-0.3,0,0	718.6±4.1 *min*: 714	706.5±6.0 *min*: 702	814.0±43.5 *min*: 743	709.4±8.4 *min*: 701	753.1±16.3 *min*: 740
LN-0,0.1,0	706.2±5.7 *min*: 690	693.2±4.7 *min*: 690	777.6±46.7 *min*: 710	693.6±4.6 *min*: 690	735.0±44.5 *min*: 706
LN-0,0.2,0	706.2±5.7 *min*: 690	692.1±3.4 *min*: 690	777.7±44.8 *min*: 719	692.8±3.6 *min*: 690	738.1±49.6 *min*: 707
LN-0,0.3,0	706.2±5.7 *min*: 690	691.5±2.4 *min*: 690	776.6±42.0 *min*: 718	692.8±3.6 *min*: 690	736.7±46.7 *min*: 708
LN-0,0,0.1	706.2±5.7 *min*: 690	692.6±3.7 *min*: 690	763.7±24.0 *min*: 721	692.0±2.6 *min*: 690	738.9±45.0 *min*: 711
LN-0,0,0.2	706.2±5.7 *min*: 690	693.0±4.8 *min*: 690	763.8±27.0 *min*: 710	694.4±5.7 *min*: 690	742.0±46.1 *min*: 712
LN-0,0,0.3	706.2±5.7 *min*: 690	691.2±1.9 *min*: 690	765.8±23.4 *min*: 724	693.2±4.1 *min*: 690	740.5±42.0 *min*: 714
LN-0.2,0.2,0	711.7±2.0 *min*: 710	702.9±5.0 *min*: 699	798.1±41.5 *min*: 728	706.8±7.1 *min*: 700	737.5±18.3 *min*: 723
LN-0.2,0,0.2	711.7±2.0 *min*: 710	704.0±6.4 *min*: 699	788.3±24.7 *min*: 737	704.2±5.0 *min*: 700	741.5±16.5 *min*: 729
LN-0,0.2,0.2	706.2±5.7 *min*: 690	691.8±2.9 *min*: 690	762.3±22.0 *min*: 723	692.0±2.6 *min*: 690	743.0±44.2 *min*: 714
LN-0.2,0.2,0.2	711.7±2.0 *min*: 710	703.8±5.7 *min*: 700	788.7±22.1 *min*: 740	705.4±5.7 *min*: 701	737.4±19.2 *min*: 726
LN-0.3,0.3,0.3	718.6±4.1 *min*: 714	706.6±6.5 *min*: 702	806.3±23.8 *min*: 749	710.4±8.4 *min*: 702	753.2±16.0 *min*: 745
U-0.2,0.2,0.2	704.8±3.1 *min*: 702	696.0±3.7 *min*: 693	773.6±24.3 *min*: 724	699.1±4.6 *min*: 693	731.6±20.3 *min*: 716
U-0.3,0.3,0.3	709.5±3.7 *min*: 706	699.9±5.0 *min*: 696	782.0±26.6 *min*: 722	701.5±5.4 *min*: 694	733.2±21.0 *min*: 722

**Table 9:** Results for the energy minimization objective across all test scenarios. Objective values are aggregated over all 108 evaluations, yielding a single score for each PF. Each cell reports the mean ± standard deviation across the ten independently evolved PFs on the first line and the minimum obtained value on the second line. Best mean results are shown in bold.

Test Type	Serial	Semi-Parallel	Parallel	Semi-Parallel-B	Parallel-B
DET-0,0,0	112,247±2270 *min*: 109,483	145,549±50,294 *min*: 123,021	152,109±33,920 *min*: 126,379	154,240±80,537 *min*: 122,341	208,250±102,861 *min*: 125,145
LN-0.1,0,0	112,039±2676 *min*: 108,269	129,853±10,370 *min*: 122,821	152,935±34,610 *min*: 126,186	141,168±38,538 *min*: 122,285	185,308±89,206 *min*: 127,278
LN-0.2,0,0	112,838±2776 *min*: 108,246	130,064±10,338 *min*: 123,006	153,378±34,506 *min*: 127,114	141,641±39,343 *min*: 121,558	185,888±89,019 *min*: 127,499
LN-0.3,0,0	113,277±2432 *min*: 109,163	130,090±10,260 *min*: 122,679	153,715±34,093 *min*: 127,749	141,805±39,551 *min*: 121,368	185,790±88,493 *min*: 127,488
LN-0,0.1,0	112,247±2270 *min*: 109,483	144,555±50,698 *min*: 122,101	141,132±11,780 *min*: 126,443	127,476±6949 *min*: 117,934	175,921±89,614 *min*: 126,213
LN-0,0.2,0	112,247±2270 *min*: 109,483	144,898±51,860 *min*: 121,732	141,424±10,988 *min*: 127,429	127,346±7239 *min*: 116,793	174,911±89,074 *min*: 126,252
LN-0,0.3,0	112,247±2270 *min*: 109,483	144,709±51,579 *min*: 121,763	141,767±11,113 *min*: 127,551	126,917±6542 *min*: 116,999	174,808±88,363 *min*: 125,747
LN-0,0,0.1	112,247±2270 *min*: 109,483	140,133±50,346 *min*: 121,495	153,183±36,749 *min*: 128,816	151,889±73,935 *min*: 120,255	186,094±91,177 *min*: 125,322
LN-0,0,0.2	112,247±2270 *min*: 109,483	140,043±51,422 *min*: 120,382	155,101±42,748 *min*: 129,219	152,299±75,955 *min*: 119,585	185,531±89,588 *min*: 126,187
LN-0,0,0.3	112,247±2270 *min*: 109,483	140,834±51,273 *min*: 121,541	156,435±46,683 *min*: 128,674	152,897±76,704 *min*: 120,852	185,365±89,421 *min*: 124,422
LN-0.2,0.2,0	112,838±2776 *min*: 108,246	129,327±9901 *min*: 122,185	142,399±11,068 *min*: 128,405	127,596±6633 *min*: 117,992	154,014±65,501 *min*: 127,734
LN-0.2,0,0.2	112,838±2776 *min*: 108,246	124,752±2732 *min*: 120,664	156,153±42,772 *min*: 129,837	142,507±43,312 *min*: 118,915	165,048±71,565 *min*: 127,539
LN-0,0.2,0.2	112,247±2270 *min*: 109,483	139,797±51,982 *min*: 120,511	138,785±5412 *min*: 129,272	127,719±7451 *min*: 119,642	153,192±68,084 *min*: 125,279
LN-0.2,0.2,0.2	112,838±2776 *min*: 108,246	124,632±2886 *min*: 120,198	139,879±5273 *min*: 130,533	127,653±6111 *min*: 119,934	132,474±4696 *min*: 126,960
LN-0.3,0.3,0.3	113,277±2432 *min*: 109,163	125,350±2606 *min*: 122,533	140,313±4799 *min*: 131,238	128,441±5985 *min*: 120,981	133,382±4512 *min*: 128,252
U-0.2,0.2,0.2	112,226±2775 *min*: 107,853	124,411±2450 *min*: 121,031	139,145±5617 *min*: 128,322	127,811±7068 *min*: 119,918	131,530±5601 *min*: 125,738
U-0.3,0.3,0.3	112,807±2780 *min*: 108,220	124,593±2242 *min*: 122,534	139,344±5512 *min*: 129,172	127,395±6784 *min*: 119,287	132,269±4942 *min*: 126,633

**Table 10:** Results for the tardiness minimization objective across all test scenarios. Objective values are aggregated over all 108 evaluations, yielding a single score for each PF. Each cell reports the mean ± standard deviation across the ten independently evolved PFs on the first line and the minimum obtained value on the second line. Best mean results are shown in bold.

Test Type	Serial	Semi-Parallel	Parallel	Semi-Parallel-B	Parallel-B
DET-0,0,0	3,517,394±4,911,515 *min*: 1,272,858	3,018,684±2,885,109 *min*: 1,210,110	2,315,090±2,300,109 *min*: 1,250,509	3,566,337±4,025,184 *min*: 1,308,559	1,439,120±271,802 *min*: 930,297
LN-0.1,0,0	3,475,506±4,837,029 *min*: 1,295,728	3,016,350±2,874,395 *min*: 1,206,817	1,603,646±479,085 *min*: 1,240,738	2,507,362±3,002,949 *min*: 1,219,902	1,428,824±286,188 *min*: 917,330
LN-0.2,0,0	3,504,015±4,895,805 *min*: 1,336,054	3,022,953±2,869,059 *min*: 1,215,753	1,546,361±493,140 *min*: 1,224,670	2,521,950±3,038,820 *min*: 1,199,907	1,429,511±288,066 *min*: 887,459
LN-0.3,0,0	3,550,482±4,988,065 *min*: 1,361,288	3,032,523±2,869,606 *min*: 1,242,329	1,551,372±472,270 *min*: 1,213,094	2,545,836±2,995,382 *min*: 1,229,698	1,432,600±287,750 *min*: 899,188
LN-0,0.1,0	3,537,530±4,916,739 *min*: 1,343,420	2,002,971±566,424 *min*: 1,213,993	2,370,938±2,478,455 *min*: 1,271,442	2,659,916±3,065,277 *min*: 1,283,278	1,444,139±267,299 *min*: 975,003
LN-0,0.2,0	3,539,480±4,918,295 *min*: 1,371,227	2,026,268±554,572 *min*: 1,253,590	2,368,827±2,460,497 *min*: 1,297,806	2,678,580±3,059,576 *min*: 1,287,342	1,463,359±285,930 *min*: 955,569
LN-0,0.3,0	3,581,873±4,904,132 *min*: 1,394,990	2,045,000±560,499 *min*: 1,270,528	2,409,334±2,537,330 *min*: 1,308,235	2,701,728±3,041,309 *min*: 1,370,673	1,497,089±287,480 *min*: 988,302
LN-0,0,0.1	3,560,397±4,954,960 *min*: 1,312,666	3,004,804±2,845,218 *min*: 1,177,200	2,260,626±2,417,882 *min*: 1,254,014	3,586,483±4,077,639 *min*: 1,285,101	1,448,779±279,216 *min*: 939,299
LN-0,0,0.2	3,616,232±5,061,074 *min*: 1,334,679	3,047,969±2,859,348 *min*: 1,213,344	2,297,374±2,478,369 *min*: 1,258,336	3,644,094±4,098,008 *min*: 1,315,652	1,472,615±287,035 *min*: 955,893
LN-0,0,0.3	3,723,803±5,249,914 *min*: 1,361,615	3,121,683±2,938,710 *min*: 1,265,949	2,355,746±2,474,243 *min*: 1,334,622	3,734,739±4,195,825 *min*: 1,361,115	1,532,400±296,312 *min*: 1,006,265
LN-0.2,0.2,0	3,555,869±4,922,616 *min*: 1,389,772	2,013,154±549,703 *min*: 1,244,420	1,547,006±436,455 *min*: 1,291,612	1,648,394±244,430 *min*: 1,245,622	1,456,583±300,100 *min*: 896,188
LN-0.2,0,0.2	2,022,343±372,794 *min*: 1,343,215	3,057,451±2,858,769 *min*: 1,223,754	1,471,642±222,367 *min*: 1,208,560	2,584,319±3,075,240 *min*: 1,202,368	1,469,182±289,342 *min*: 937,424
LN-0,0.2,0.2	3,651,489±5,098,453 *min*: 1,388,800	2,067,709±566,772 *min*: 1,276,945	2,334,982±2,553,197 *min*: 1,329,592	2,727,041±3,089,169 *min*: 1,323,947	1,505,385±281,620 *min*: 991,492
LN-0.2,0.2,0.2	2,059,124±342,512 *min*: 1,396,523	2,064,047±566,497 *min*: 1,267,127	1,496,135±179,914 *min*: 1,311,832	1,686,141±238,629 *min*: 1,299,616	1,498,555±305,819 *min*: 928,650
LN-0.3,0.3,0.3	2,161,896±328,529 *min*: 1,504,091	2,123,752±530,973 *min*: 1,342,451	1,574,805±192,891 *min*: 1,372,330	1,810,874±254,221 *min*: 1,352,172	1,568,568±303,623 *min*: 977,262
U-0.2,0.2,0.2	1,990,790±345,182 *min*: 1,358,960	2,003,330±564,319 *min*: 1,200,782	1,450,297±185,586 *min*: 1,222,586	1,635,515±244,100 *min*: 1,278,533	1,449,908±292,457 *min*: 892,732
U-0.3,0.3,0.3	2,018,648±351,991 *min*: 1,369,182	2,035,246±570,841 *min*: 1,253,462	1,490,303±180,828 *min*: 1,266,529	1,670,210±247,741 *min*: 1,267,831	1,469,011±282,796 *min*: 939,074

**Table 11:** Pairwise Wilcoxon signed-rank tests with Holm correction for the three objectives, based on n=1746 paired test observations. A checkmark indicates a statistically significant difference (p<0.05). Exact *p*-values are reported for nonsignificant comparisons. The median difference $\tilde{\Delta }$ is calculated as row minus column.

Objective	Method	Serial	Semi-Parallel	Parallel	Semi-Parallel-B	Parallel-B
Vehicles	Serial	–	✓ ($\tilde{\Delta }=0$)	✓ ($\tilde{\Delta }=-1$)	✓ ($\tilde{\Delta }=0$)	✓ ($\tilde{\Delta }=0$)
	Semi-parallel		–	✓ ($\tilde{\Delta }=-1$)	✓ ($\tilde{\Delta }=0$)	✓ ($\tilde{\Delta }=0$)
	Parallel			–	✓ ($\tilde{\Delta }=1$)	✓ ($\tilde{\Delta }=0$)
	Semi-parallel-B				–	✓ ($\tilde{\Delta }=0$)
	Parallel-B					–
Energy	Serial	–	✓ ($\tilde{\Delta }=-57.55$)	✓ ($\tilde{\Delta }=-186.22$)	✓ ($\tilde{\Delta }=-70.81$)	✓ ($\tilde{\Delta }=-143.39$)
	Semi-parallel		–	✓ ($\tilde{\Delta }=-129.60$)	✓ ($\tilde{\Delta }=-12.05$)	✓ ($\tilde{\Delta }=-75.68$)
	Parallel			–	✓ ($\tilde{\Delta }=111.77$)	✓ ($\tilde{\Delta }=46.23$)
	Semi-parallel-B				–	✓ ($\tilde{\Delta }=-57.48$)
	Parallel-B					–
Tardiness	Serial	–	✓ ($\tilde{\Delta }=61.86$)	✓ ($\tilde{\Delta }=3117.92$)	✓ ($\tilde{\Delta }=2008.03$)	✓ ($\tilde{\Delta }=3157.16$)
	Semi-parallel		–	✓ ($\tilde{\Delta }=2833.24$)	✓ ($\tilde{\Delta }=1770.64$)	✓ ($\tilde{\Delta }=2851.22$)
	Parallel			–	✓ ($\tilde{\Delta }=-1753.00$)	p=0.289 ($\tilde{\Delta }=-158.55$)
	Semi-parallel-B				–	✓ ($\tilde{\Delta }=1425.74$)
	Parallel-B					–

**Table 12:** Median tree size and depth, with minimum and maximum values in brackets, for the best-of-run priority functions obtained across ten independent GP runs.

RGS	Vehicle Minimization		Energy Minimization		Tardiness Minimization	
	Nodes	Depth	Nodes	Depth	Nodes	Depth
Serial	4 [2–27]	2 [1–8]	26 [2–275]	9.5 [1–69]	138.5 [23–318]	22 [8–35]
Semi-parallel	3 [2–4]	1 [1–3]	25.5 [6–272]	10 [2–40]	105 [38–315]	22 [10–54]
Semi-parallel-B	3 [2–5]	1 [1–2]	43 [4–167]	9 [3–24]	63.5 [9–145]	12.5 [4–36]
Parallel	20.5 [2–193]	7 [1–22]	48 [2–178]	13 [1–32]	63 [18–327]	11.5 [8–51]
Parallel-B	8.5 [2–43]	4 [1–11]	51 [25–120]	13 [8–32]	22.5 [15–82]	9 [5–23]

**Table 13:** Support of local semantic patterns in the best-of-run priority functions. Each value reports the number of supporting trees among ten independent GP runs.

Objective and Semantic Pattern	Serial	Semi-Parallel	Semi-Parallel-B	Parallel	Parallel-B
Vehicle: demand–visit energy ($D_{ni}+E_{ni}$), local	0/10	7/10	6/10	5/10	6/10
Vehicle: demand–visit energy ($D_{ni}+E_{ni}$), direct	0/10	6/10	6/10	0/10	2/10
Energy: visit energy + depot/charging energy ($E_{ni}+R$), local	3/10	6/10	1/10	4/10	2/10
Tardiness: energy + temporal/urgency signals (E+T), local	8/10	8/10	9/10	9/10	6/10
Tardiness: visit energy + temporal/urgency signals ($E_{ni}+T$), local	6/10	5/10	8/10	9/10	3/10
Tardiness: energy + stochastic-variability signals (E+S), local	1/10	5/10	6/10	2/10	0/10

*Notes:* E denotes the set of energy-related terminals $\left\{E_{ni},EC_{ni},EDep_{ni},ERP_{ni},EDep_{pvk},ERP_{pvk},E_{vk}\right\}$. T denotes temporal and urgency-related terminals $\left\{DD_{ni},RT_{ni},ST_{ni},T_{vk},{\mathrm{Slack}}_{TW},{\mathrm{Slack}}_{Self}\right\}$. R denotes terminals measuring the energy required to reach the depot or nearest charging station from either the candidate customer or the vehicle’s current position: $\left\{EDep_{ni},ERP_{ni},EDep_{pvk},ERP_{pvk}\right\}$. S denotes stochastic-variability descriptors $\left\{\mathrm{Var}\text{\_}D_{ni},\mathrm{Var}\text{\_}S_{ni},\mathrm{Var}\text{\_}T_{ij}\right\}$. A local pattern requires the participating signals to occur within two edges of their lowest common ancestor. A direct pattern requires them to be sibling leaves of the same function node. Each tree contributes at most once to the support of a given pattern.

**Figure A1:**
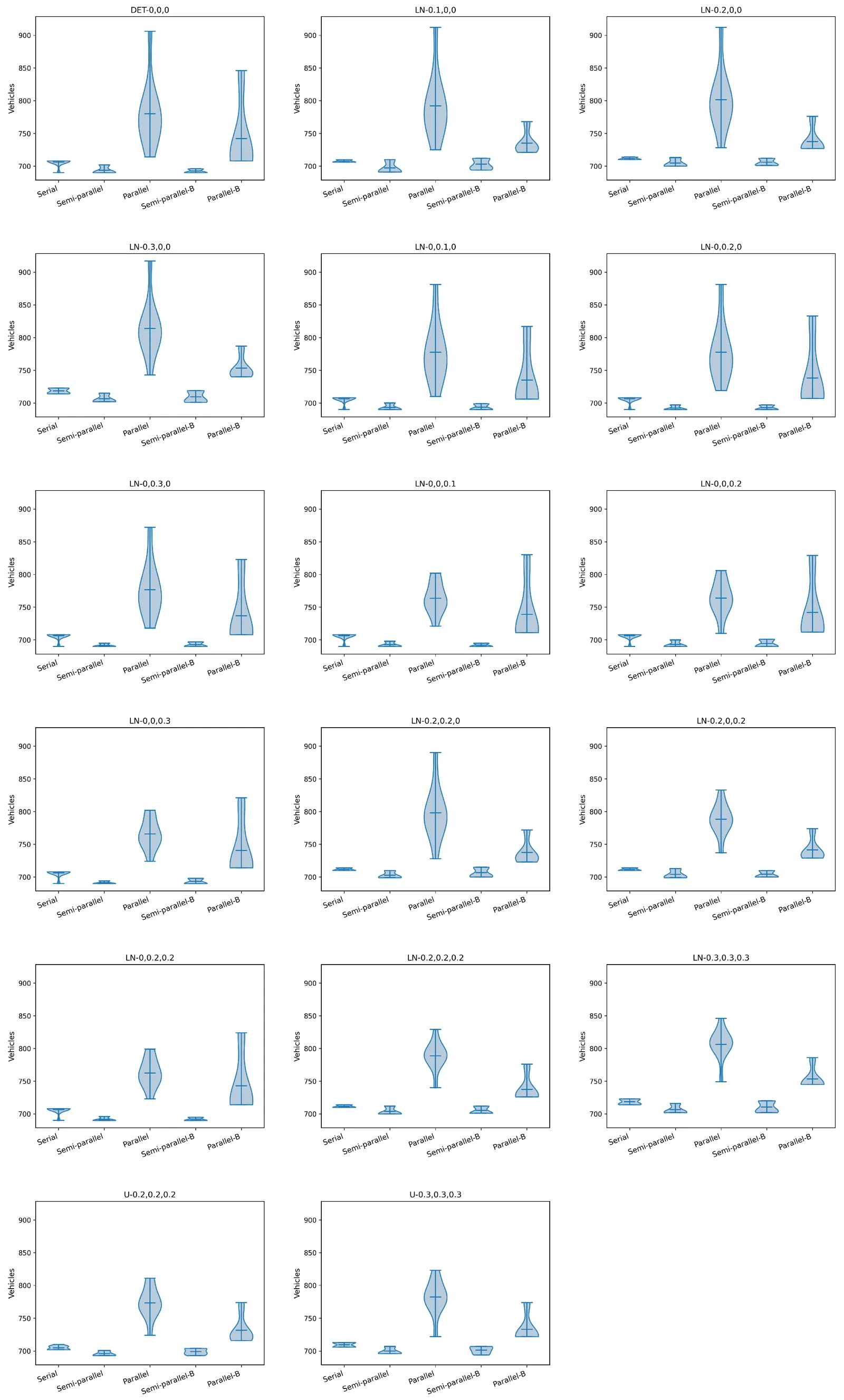
Scenario-wise violin plots for the vehicle minimization objective. Each subplot corresponds to one experimental scenario and shows the distribution of the number of vehicles obtained for the five route generation schemes over all runs.

**Figure A2:**
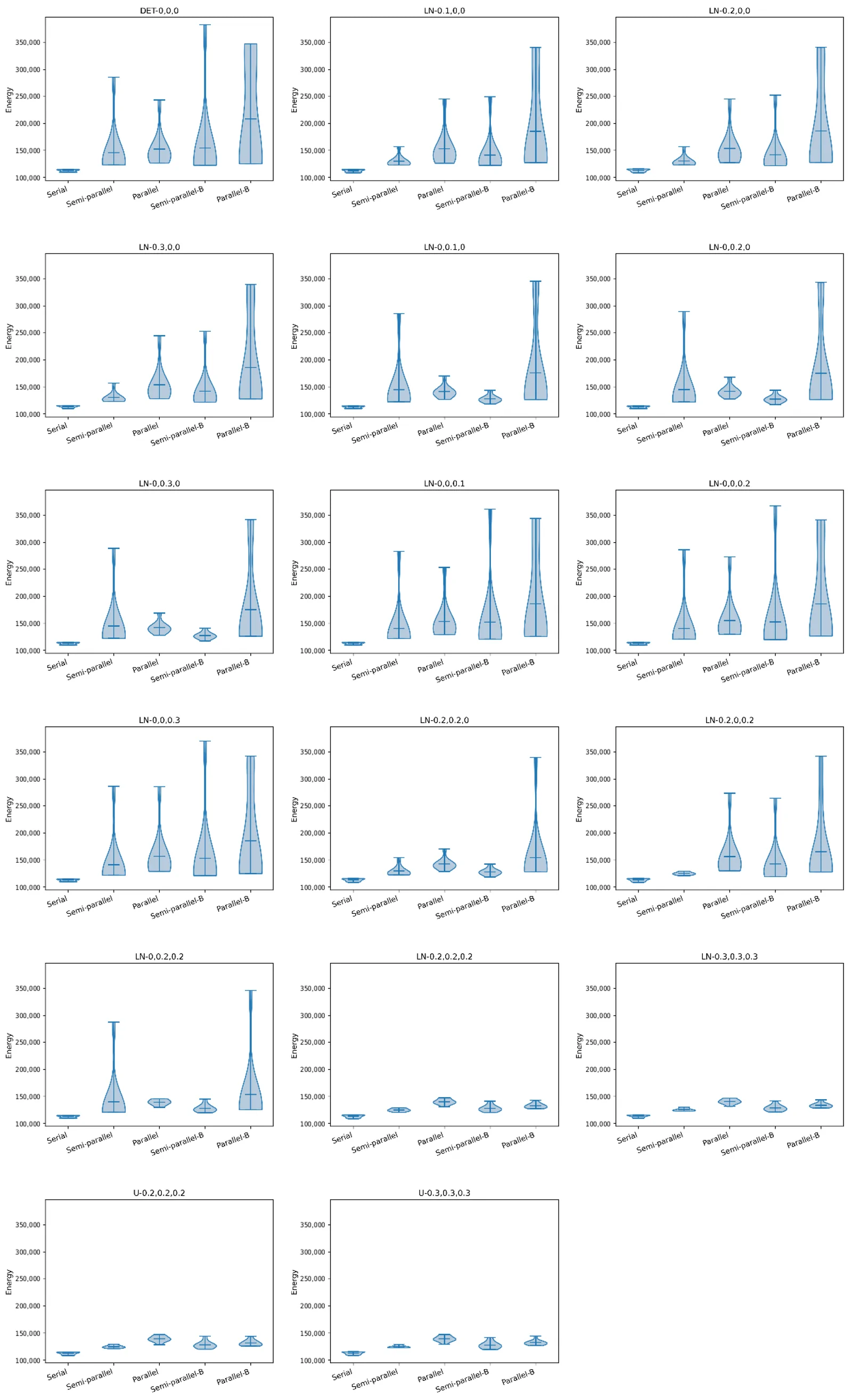
Scenario-wise violin plots for the energy minimization objective. Each subplot corresponds to one experimental scenario and shows the distribution of energy consumption obtained for the five route generation schemes over all runs.

**Figure A3:**
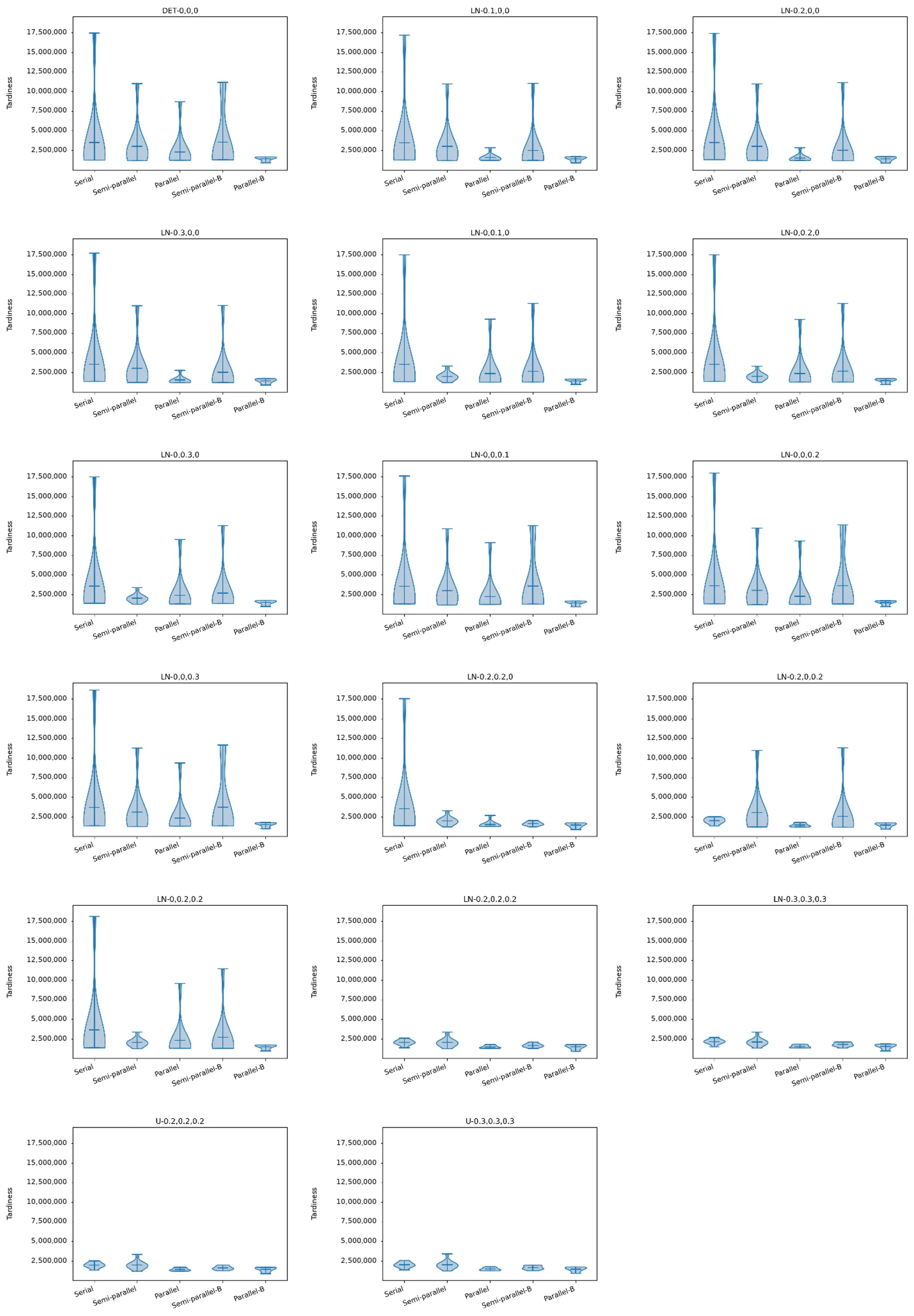
Scenario-wise violin plots for the tardiness minimization objective. Each subplot corresponds to one experimental scenario and shows the distribution of tardiness values obtained for the five route generation schemes over all runs.

## Data Availability

The data presented in this study are available in repository at https://github.com/AlanDurdevic/stochastic-EVRP-GP/tree/main/data/stohastic (accessed on 8 September 2026). These data were derived from the following resources available in the public domain: https://data.mendeley.com/datasets/h3mrm5dhxw/1 (accessed on 8 September 2026).

## Abbreviations

The following abbreviations are used in this manuscript:

VRP	Vehicle Routing Problem
VRPTW	Vehicle Routing Problem with Time Windows
EVRP	Electric Vehicle Routing Problem
EVRPTW	Electric Vehicle Routing Problem with Time Windows
RGS	Route Generation Scheme
PF	Priority Function
GP	Genetic Programming
ALNS	Adaptive Large Neighborhood Search
ILS	Iterated Local Search
VNS	Variable Neighborhood Search

## Appendix A. Scenario-Wise Distribution of Objective Values

This appendix provides a more detailed view of the experimental results through scenario-wise violin plots for all three optimization objectives. Unlike the aggregated plots presented in the main text, these figures show the distribution of results separately for each test scenario and for each route generation scheme. In this way, they provide additional insight into the variability, spread, and shape of the obtained outcomes under different stochastic settings.
